# A Bayesian model selection approach for identifying differentially expressed transcripts from RNA sequencing data

**DOI:** 10.1111/rssc.12213

**Published:** 2017-02-07

**Authors:** Panagiotis Papastamoulis, Magnus Rattray

**Affiliations:** ^1^ University of Manchester UK

**Keywords:** Collapsed Gibbs sampler, Mixture models, Reversible jump Markov chain Monte Carlo sampling, Ribonucleic acid sequencing

## Abstract

Recent advances in molecular biology allow the quantification of the transcriptome and scoring transcripts as differentially or equally expressed between two biological conditions. Although these two tasks are closely linked, the available inference methods treat them separately: a primary model is used to estimate expression and its output is post processed by using a differential expression model. In the paper, both issues are simultaneously addressed by proposing the joint estimation of expression levels and differential expression: the unknown relative abundance of each transcript can either be equal or not between two conditions. A hierarchical Bayesian model builds on the BitSeq framework and the posterior distribution of transcript expression and differential expression is inferred by using Markov chain Monte Carlo sampling. It is shown that the model proposed enjoys conjugacy for fixed dimension variables; thus the full conditional distributions are analytically derived. Two samplers are constructed, a reversible jump Markov chain Monte Carlo sampler and a collapsed Gibbs sampler, and the latter is found to perform better. A cluster representation of the aligned reads to the transcriptome is introduced, allowing parallel estimation of the marginal posterior distribution of subsets of transcripts under reasonable computing time. Under a fixed prior probability of differential expression the clusterwise sampler has the same marginal posterior distributions as the raw sampler, but a more general prior structure is also employed. The algorithm proposed is benchmarked against alternative methods by using synthetic data sets and applied to real RNA sequencing data. Source code is available on line from https://github.com/mqbssppe/cjBitSeq.

## 1 Introduction

Quantifying the transcriptome of a given organism or cell is a fundamental task in molecular biology. Ribonucleic acid sequencing (which is known as ‘RNA‐seq’) technology produces transcriptomic data in the form of short reads (Mortazavi *et al*., 2008). These reads can be used either to reconstruct the transcriptome by using *de novo* or guided assembly, or to estimate the abundance of known transcripts given a reference annotation. Here, we consider the latter scenario in which transcripts are defined by annotation. In such a case, millions of short reads are aligned to the reference transcriptome (or genome) by using mapping tools such as ‘Bowtie’ (Langmead *et al*., 2009) (or ‘TopHat’ (Trapnell *et al*., 2009)). Of particular interest is the identification of differentially expressed transcripts (or isoforms) across different samples. Throughout this paper the term transcript refers to isoforms, so differential transcript detection has the same meaning as differential isoform detection. Most genes in higher eukaryotes can be spliced into alternative transcripts that share specific parts of their nucleotide sequence. Thus, a short read is not uniquely aligned to the transcriptome and its origin remains uncertain, making transcript expression estimation non‐trivial. Probabilistic models provide a powerful means to estimate transcript abundances as they can take this ambiguous read assignment into consideration in a principled manner.

There are numerous methods that estimate transcript expression from RNA‐seq data, including ‘RNA‐seq by expectation–maximization’ (Li and Dewey, 2011), ‘isoform estimation by expectation–maximization’ (Nicolae *et al*., 2011), ‘Cufflinks’ (Trapnell *et al*., 2013, 2010), ‘BitSeq’ (stage 1) (Glaus *et al*., 2012), ‘transcript isoform estimation with gapped alignment of RNA‐seq data’ (Nariai *et al*., 2013) and ‘Casper’ (Rossell *et al*., 2014). Some of these methods also include a second stage for performing differential expression (DE) analysis at the transcript level (e.g. ‘Cuffdiff’ and BitSeq stage 2) and stand‐alone methods for transcript level DE calling have also been developed such as ‘EBSeq’ (Leng *et al*., 2013) and ‘MetaDiff’ (Jia *et al*., 2015). Cuffdiff uses an asymptotically normal test statistic by applying the delta method to the log‐ratio of transcript abundances between two samples, given the estimated expression levels using Cufflinks. EBSeq estimates the Bayes factor of a model under DE or non‐DE for each transcript, building a negative binomial model on the estimated read counts from any method. BitSeq stage 2 ranks transcripts as differentially expressed by the probability of positive log‐ratio based on the Markov chain Monte Carlo MCMC output from BitSeq stage 1, which estimates the expression levels by assuming a mixture model. Gene level DE analysis is also available by using count‐based methods such as ‘edgeR’ (Robinson *et al*., 2010) and ‘DESeq’ (Anders and Huber, 2010) but here we limit our attention to methods that are designed for transcript level DE calling.

All existing methods for transcript level DE calling apply a two‐step procedure. The mapped RNA‐seq data are used as input of a first‐stage analysis to estimate transcript expression. The output of this stage is then post processed at a second stage to classify transcripts as differentially expressed or non‐differentially expressed. The bridge between the two stages is based on certain parametric assumptions for the distribution of the estimates of the first‐stage and/or the use of asymptotic results (as previously described above). Also, transcript level expression estimates are correlated through sharing of reads and this correlation is typically ignored in the second stage. Such two‐stage approaches are quite useful in practice since the DE question is not always the main aim of the analysis; therefore estimating expression is useful in itself. However, when the main purpose of an experiment is DE calling then the two‐stage procedure increases the modelling complexity and may result in overfitting, since there is no guarantee that the underlying assumptions are valid. Note that a recent method (Gu *et al*., 2014) addresses the joint estimation of expression and DE modelling of exon counts under a Bayesian approach but at the gene level rather than the transcript level that is considered here.

The contribution of this paper is to develop a method for the joint estimation of expression and DE at the transcript level. The method builds on the Bayesian framework of the BitSeq (stage 1) model where transcript expression estimation reduces to estimating the posterior distribution of the weights of a mixture model by using MCMC sampling (Glaus *et al*., 2012). The novelty in the present study is that DE is addressed by inferring which weights differ between two mixture models. This is achieved by using two samplers. A reversible jump MCMC (RJMCMC) algorithm (Green, 1995) updates both transcript expression and DE parameters, and a collapsed Gibbs algorithm is developed which avoids transdimensional transitions. The high dimensional setting of RNA‐seq data studies makes the convergence to the joint posterior distribution computationally challenging. To alleviate this computational burden and to allow easier parallelization, a new cluster representation of the transcriptome is introduced which collapses the problem to subsets of transcripts sharing aligned reads.

The rest of the paper is organized as follows. The mixture model that is used in the original BitSeq set‐up is reviewed in Section 2.1. The prior assumptions of the new (clusterwise joint BitSeq) model called cjBitSeq is introduced in Section 2.2. The full conditional distributions are given in Section 2.3 and two MCMC samplers are described in Section 2.4. A cluster representation of aligned reads and transcripts is discussed in Section 2.5 and details over false discovery rate (FDR) estimation are given in Section 2.6. Large‐scale simulation studies are presented in Section 3.2 and the method proposed is illustrated on a real human data set in Section 3.3. The paper concludes in Section 4 with a synopsis and discussion.

## 2 Methods

In the BitSeq model, the mixture components correspond to annotated transcript sequences and the mixture weights correspond to their relative expression levels. The data likelihood is then computed by considering the alignment of reads (or read pairs) against each mixture component. Essentially, this model is modified here to construct a well‐defined probability of DE or non‐DE when two samples are available.

We induce a set of free parameters of varying dimension, depending on the number of different weights between two mixture models. Assuming two independent Dirichlet prior distributions, the Gibbs sampler (Geman and Geman, 1984; Gelfand and Smith, 1990) draws samples from the full conditionals, which are independent Dirichlet and generalized Dirichlet (Connor and Mosimann, 1969; Wong, 1998, 2010) distributions. This representation allows the integration of the corresponding parameters as stated in theorem 2. Therefore, we provide two MCMC samplers depending on whether transcript expression levels are integrated out or not. These samplers converge to the same target distribution but using different steps to update the state of each transcript: the first uses a birth–death move type (Richardson and Green, 1997; Papastamoulis and Iliopoulos, 2009) and the second is a block update from the full conditional distribution. After detecting clusters of transcripts and reads, it is shown that the parallel application of the algorithm to each cluster converges to proper marginals of the full posterior distribution.

### 2.1 BitSeq

Let **x**=(*x*_1_,…,*x*_*r*_), $x_{i}\in X$, *i*=1,…,*r*, denote a sample of *r* short reads aligned to a given set of *K* transcripts. The sample space X consists of all sequences of letters A, C, G and T. Assuming that reads are independent, the joint probability density function of the data is written as

(1) $$x|\theta ∼\prod_{i=1}^{r}\sum_{k=1}^{K}\theta_{k}f_{k}\left(x_{i}\right).$$

The number of components, *K*, is equal to the number of transcripts and it is considered as known since the transcriptome is given. The parameter vector $\theta =\left(\theta_{1},\ldots ,\theta_{K}\right)\in P_{K-1}$ denotes relative abundances, where

$$P_{K-1}:=\left\{p_{k}⩾0,k=1,\ldots ,K-1:\sum_{k=1}^{K-1}p_{k}⩽1;p_{K}:=1-\sum_{k=1}^{K-1}p_{k}\right\}.$$

The component‐specific density *f*_*k*_(·) corresponds to the probability of a read aligning at some position of transcript *k*,* k*=1,…,*K*. Since we assume a known transcriptome, ${\left\{f_{k}\right\}}_{k=1}^{K}$ are known as well and they are computed according to the methodology that is described in Glaus *et al*. (2012) (see also appendix A in the on‐line supplementary material), taking into account position and sequence‐specific bias correction methods.

*A priori* it is assumed that $\theta ∼D_{K-1}\left(\alpha_{1},\ldots ,\alpha_{K}\right)$, with $D_{j}$ denoting the Dirichlet distribution defined over $P_{j}$. Furthermore, it is assumed that *α*_1_=  …=*α*_*K*_=1, which is equivalent to the uniform distribution in $P_{K-1}$. In the original implementation of BitSeq (Glaus *et al*., 2012), MCMC samples are drawn from the posterior distribution of ***θ***|**x** by using the Gibbs sampler whereas more recently variational Bayes approximations have also been included for faster inference (Papastamoulis *et al*., 2014; Hensman *et al*., 2015).

Given the output of BitSeq stage 1 for two different samples, BitSeq stage 2 implements a one‐sided test, probability of positive log‐ratio, PPLR, for DE analysis. However, this approach does not define transcripts as differentially expressed or non‐differentially expressed and is therefore not directly comparable with standard two‐sided tests that are available in most other packages (Trapnell *et al*., 2013; Leng *et al*., 2013). Also, correlations between transcripts in the posterior distribution for each sample are discarded during the DE stage, leading to potential loss of accuracy when making inferences. To deal with these limitations, a new method for performing DE analysis is presented next.

### 2.2 cjBitSeq

Assume that we have at hand two samples **x**:=(*x*_1_,…,*x*_*r*_) and **y**:=(*y*_1_,…,*y*_*s*_) denoting the number of (mapped) reads for sample **x** and **y** respectively. Now, let *θ*_*k*_ and *w*_*k*_ denote the unknown relative abundance of transcript *k*=1,…,*K* in sample **x** and **y** respectively. Define the parameter vector of relative abundances as $\theta =\left(\theta_{1},\ldots ,\theta_{K-1};\theta_{K}\right)\in P_{K-1}$ and $w=\left(w_{1},\ldots ,w_{K-1};w_{K}\right)\in P_{K-1}$. Under the standard BitSeq model the prior on the parameters ***θ*** and **w** would be a product of independent Dirichlet distributions. In this case the probability *θ*_*k*_=*w*_*k*_ under the prior is 0 and it is not straightforward to define non‐differentially expressed transcripts. To model DE we would instead like to identify instances where transcript expression has not changed between samples. Therefore, we introduce a non‐zero probability for the event *θ*_*k*_=*w*_*k*_. This leads us to define a new model with a non‐independent prior for the parameters ***θ*** and **w**.

Definition 1 (state vector.) Let $c:=(c_{1},\ldots ,c_{K})\in C$, where C is the set defined by

- $c_{k}\in \left\{0,1\right\}$, *k*=1,…,*K*,
- $c_{+}:=\Sigma_{k=1}^{K}c_{k}\neq 1$.

Then, for *k*=1,…,*K* let

$$\left\{\begin{array}{cc} \theta_{k}=w_{k}, & \text{if}\;c_{k}=0,\\ \theta_{k}\neq w_{k}, & \text{if}\;c_{k}=1. \end{array}\right.$$

We shall refer to vector *c* as the state vector of the model.

For example, assume that *K*=6 and *c*=(1,0,0,1,0,1). According to definition 1, *θ*_*k*_=*w*_*k*_ for *k*=2,3,5 and *θ*_*k*_≠*w*_*k*_ for *k*=1,4,6. From definition 1 it is obvious that the sum of the elements in *c* cannot be equal to 1 because either all *θ*s must be equal to *w*s, or at least two of them must be different. The introduction of such dependences between the elements of ***θ*** and **w** has non‐trivial effects on the prior assumptions of course. It is clear that with this approach we should define a valid conditional prior distribution for ***θ***,**w**|*c*.

First we impose a prior assumption on *c*. We shall consider the Jeffreys (Jeffreys, 1946) prior distribution for a Bernoulli trial, i.e. $P(c_{k}=1|\pi )=\pi$ with *π* following a beta distribution. Since $c_{+}\neq 1$, the prior distribution of the state vector *c* is expressed as

(2) $$\pi ∼\text{beta}(\frac{1}{2},\frac{1}{2}),$$

(3) $$P\left(c|\pi \right)=P\left(c|c_{+}\neq 1,\pi \right)=\frac{\pi^{c_{+}}{\left(1-\pi \right)}^{K-c_{+}}}{1-K\pi {\left(1-\pi \right)}^{K-1}},c\in C.$$

Next we proceed to the definition of a proper prior structure for the weights of the mixture. At this step extra care should be taken for everything to make sense as a probabilistic space. It is obvious that (***θ***,**w**) should be defined conditionally on the state vector *c*. What it is less obvious is that (***θ***,**w**) should be defined conditionally on a parameter of varying dimension. At this point, we introduce some extra notation.

Definition 2 (dead and alive subsets and permutation of the labels.) For a given state vector *c*, define the order‐specific subsets

$$C_{0}\left(c\right):=\left\{\tau_{1}<\;\;\ldots <\tau_{K-c_{+}}\in \left\{1,\ldots ,K\right\}:c_{\tau_{k}}=0\;\forall \;k=1,\ldots ,K-c_{+}\right\}$$

and

$$C_{1}\left(c\right):=\left\{\tau_{K-c_{+}+1}<\;\;\ldots <\tau_{K}\in \left\{1,\ldots ,K\right\}:c_{\tau_{k}}=1\;\forall \;k=K-c_{+}+1,\ldots ,K\right\}.$$

These sets will be called dead and alive subsets of the transcriptome index respectively. Moreover, *τ*=(*τ*_1_,…,*τ*_*K*_) denotes the unique permutation of {1,…,*K*} obeying the ordering within the dead and alive subsets.

As will be made clear later, it is convenient to define a unique labelling within the dead and alive subsets so we also explicitly define the corresponding permutation *τ* of the labels. To clarify definition 2, assume that *c*=(1,0,0,1,0,1). Then definition 2 implies that *C*_0_(*c*)={2,3,5}, *C*_1_(*c*)={1,4,6} and *τ*=(2,3,5,1,4,6). The order‐specific definition of these subsets excludes {3,2,5} (for example) from the definition of a dead subset.

It is clear that, if *C*_0_(*c*)=∅, then both ***θ*** and **w** have *K*−1 free parameters each. However, if *C*_0_(*c*)≠∅, the free parameters lie in a lower dimensional space. This means that (***θ***,**w**) should be defined given *c* by taking into account the set of free parameters that are actually allowed by the state vector. In particular, (***θ***,**w**) are pseudoparameters. The actual parameters of our problem are defined in lemma 1.

In what follows, the notation *τ* ***σ*** should be interpreted as the reordering of vector ***σ***=(*σ*_1_,…,*σ*_*K*_) under permutation *τ*. For example, assume that *τ*=(3,1,2) and ***σ***=(*σ*_1_,*σ*_2_,*σ*_3_); then *τ* ***σ***=(*σ*_3_,*σ*_1_,*σ*_2_). Let also *τ* ^−1^ denote the inverse permutation of *τ*.

Lemma 1 (existence and uniqueness of free parameters.) For every (*c*,*τ*,***θ***,**w**) respecting definitions 1 and 2 there is a unique set of free parameters:

(4) $$\left(u,v\right)\in P_{K-1}\times P_{c_{+}-1},$$

such that

(5) $$\theta =\tau^{-1}u,$$

(6) $$w=\tau^{-1}\varpi ,$$

where $\varpi =(\left\{u_{\tau_{k}^{-1}}:k\in C_{0}\left(c\right)\right\},v\Sigma_{k\in C_{1}\left(c\right)}u_{\tau_{k}^{-1}})$ under the conventions $P_{-1}:=\emptyset$ and $\emptyset \;\Sigma_{k\in \emptyset }\;u_{k}:=\emptyset$.

It is trivial to show that (*c*,*τ*,**u**,**v**)→(***θ***,**w**) is a ‘one‐to‐one’ and ‘onto’ mapping (bijective function).

For example, assume that *c*=(1,0,0,1,0,1), where *C*_0_(*c*)={2,3,5} and *C*_1_(*c*)={1,4,6}. Then, *τ*=(2,3,5,1,4,6) and *τ* ^−1^=(4,1,2,5,3,6). According to state *c* we should have that *θ*_2_=*w*_2_, *θ*_3_=*w*_3_ and *θ*_5_=*w*_5_, whereas *θ*_*k*_≠*w*_*k*_ for $k\in C_{1}\left(c\right)$. Lemma 1 states that ***θ*** and **w** can be expressed as a transformation of two independent parameters: $u=\left(u_{1},u_{2},u_{3},u_{4},u_{5},u_{6}\right)\in P_{5}$ and $v=\left(v_{1},v_{2},v_{3}\right)\in P_{2}$. According to equation (5), ***θ*** is a permutation of the vector **u**:

$$\theta |\left(c,u\right)=\left(u_{4},u_{1},u_{2},u_{5},u_{3},u_{6}\right).$$

Next, **w** is obtained by a permutation of ***ϖ***, which is a linear transformation of **u** and **v**, i.e. ***ϖ***=(*u*_1_,*u*_2_,*u*_3_,*v*_1_(*u*_4_+*u*_5_+*u*_6_),*v*_2_(*u*_4_+*u*_5_+*u*_6_),*v*_3_(*u*_4_+*u*_5_+*u*_6_)). According to equation (6),

$$w|\left(c,u,v\right)=\left(v_{1}\left(u_{4}+u_{5}+u_{6}\right),u_{1},u_{2},v_{2}\left(u_{4}+u_{5}+u_{6}\right),u_{3},v_{3}\left(u_{4}+u_{5}+u_{6}\right)\right)\;\;.$$

Comparing the last two expressions for ***θ*** and **w**, it is obvious that *θ*_2_=*w*_2_, *θ*_3_=*w*_3_ and *θ*_5_=*w*_5_, whereas *θ*_*k*_≠*w*_*k*_ for all remaining entries, which is the configuration that is implied by the state vector *c*. Note also that $\left\{u_{\tau_{k}^{-1}};k\in C_{0}\left(c\right)\right\}=\left(u_{1},\ldots ,u_{K-c_{+}}\right)$ and $\left\{u_{\tau_{k}^{-1}};k\in C_{1}\left(c\right)\right\}=\left(u_{K-c_{+}+1},\ldots ,u_{K}\right)$ and $\Sigma_{k\in C_{1}\left(c\right)}w_{k}=\Sigma_{k\in C_{1}\left(c\right)}\theta_{k}=\Sigma_{k\in C_{1}\left(c\right)}u_{\tau_{k}^{-1}}$.

Now, it should be clear that given a state vector *c*, as well as the independent free parameters **u** and **v**, the pseudoparameters ***θ*** and **w** are deterministically defined. In other words, the conditional distributions of ***θ*** and **w** are Dirac distributions, gathering all their probability mass into the single points defined by equations (5) and (6). Hence, the conditional prior distribution for transcript expression is written as

(7) $$f\left(\theta ,w|c,\tau ,u,v\right)=1_{\theta ,\;w}\left[\left\{\theta \left(c,\tau ,u\right),w\left(c,\tau ,u,v\right)\right\}\right],$$

with ***θ***(*c*,*τ*,**u**) and **w**(*c*,*τ*,**u**,**v**) as in equations (5) and (6) respectively.

Moreover, we stress that, if the permutation *τ* were not uniquely defined according to definition 2, then we would have had to take into account all the possible permutations within the dead and alive subsets. However, such an approach would lead to an increased modelling complexity without making any difference on the inference. That said, the conditional prior distribution of *τ* given *c* is Dirac:

(8) $$f\left(\tau |c\right)=1_{\tau }\left\{\tau \left(c\right)\right\},$$

where *τ*(*c*) denotes the unique permutation (given *c*) in definition 2.

At this point we state our prior assumptions for the free parameters, given a state vector *c*. We assume that *a priori* **u** and **v** are independent random variables distributed according to a Dirichlet distribution, i.e.

(9) $$u|c∼D_{K-1}\;\left(\alpha_{1},\ldots ,\alpha_{K}\right),$$

(10) $$v|c∼D_{c_{+}-1}\;\left(\gamma_{1},\ldots ,\gamma_{c_{+}}\right).$$

In the applications, we shall furthermore assume that *α*_*k*_=1 for all *k*=1,…,*K* and *γ*_*l*_=1 for all $l=1,\ldots ,c_{+}$, to assign a uniform prior distribution over $P_{K-1}\times P_{c_{+}-1}$. Now, the following theorem holds.

Theorem 1 Assume that distributions (9) and (10) hold true and furthermore *α*_*k*_=*γ*_*k*_=*α* for all *k*=1,…,*K*. Then, ***θ*** and **w** are marginally identical random variables following the $D_{K-1}\left(\alpha ,\ldots ,\alpha \right)$ distribution.

For a proof of theorem 1, see appendix C in the on‐line supplementary material.

**Figure 1:**
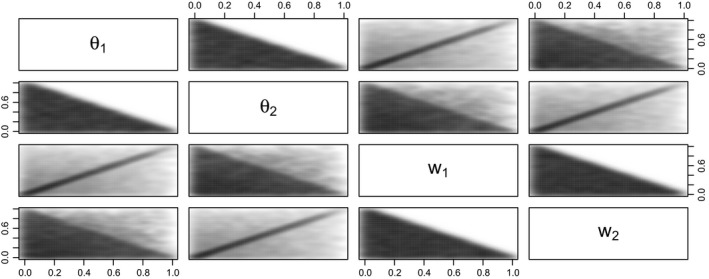
Simulation from the prior distribution (7) of (***θ***,**w**) for *K*=3 and *α*_*k*_=*γ*_*k*_=1 for *k*=1,2,3, and also assuming the Jeffreys prior for *c*: theorem 1 states that, marginally, θ∼D(1,1,1) and w∼D(1,1,1)

Note here that theorem 1 does not imply that ***θ*** and **w** are *a priori* independent. As shown in Fig. 1, *θ*_*k*_ is exactly equal to *w*_*k*_ with probability *P*(*c*_*k*_=0)>0, *k*=1,…,*K*.

The model definition is completed by considering the latent allocation variables of the mixture model. Let ***ξ***={*ξ*_1_,…,*ξ*_*r*_} and **z**={*z*_1_,…,*z*_*s*_} with

$$\begin{array}{cc} & P\left(\xi_{i}=k|\theta \right)=\theta_{k},\;\text{independent for}\;i=1,\ldots ,r,\\ & P\left(z_{j}=k|w\right)=w_{k},\;\text{independent for}\;j=1,\ldots ,s, \end{array}$$

for *k*=1,…,*K*. Moreover, ***ξ*** and **z** are assumed conditionally independent given ***θ*** and **w**, i.e. *P*(***ξ***,**z**|***θ***,**w**)=*P*(***ξ***|***θ***)*P*(**z**|**w**). Now, the joint distribution of the complete data (**x**,**y**,***ξ***,**z**) factorizes as follows:

(11) $$f\left(x,y,\xi ,z|\theta ,w\right)=\prod_{i=1}^{r}\theta_{\xi_{i}}f_{\xi_{i}}\left(x_{i}\right)\prod_{j=1}^{s}w_{z_{j}}f_{z_{j}}\left(y_{j}\right).$$

Let **g**=(**x**,**y**,***ξ***,**z**,***θ***,**w**,**u**,**v**,*c*,*τ*,*π*). From equations (2), (3) and (7), (8), (9), (10), (11), the joint distribution of **g** is defined as

(12) f(g|α,γ,K)=f(x,y,ξ,z|θ,w)f(u|α,K)f(v|c,γ)f(θ|τ,u)f(w|c,τ,u,v)f(τ|c)f(c|K,π)f(π).

**Figure 2:**
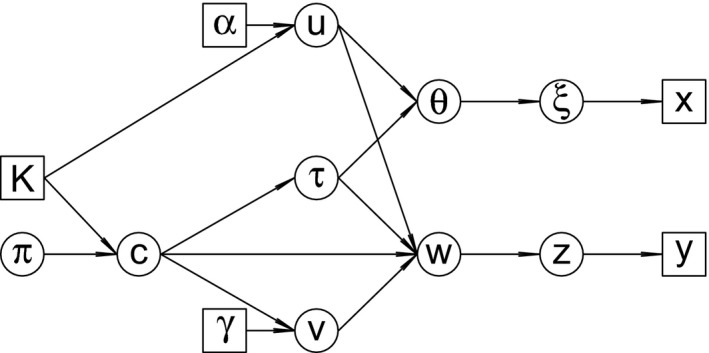
Directed acyclic graph representation of the hierarchical model (12)

Equation (12) defines a hierarchical model whose graphical representation is given in Fig. 2 with circles and squares denoting respectively unobserved and observed or known variables.

### 2.3 Full conditional distributions for the Gibbs updates

In this section, the full conditional distributions are derived. Let *h*|⋯ denote the conditional distribution of a random variable *h* given the values of the rest of the variables. We also denote by **x**_[−*i*]_ all remaining members of a generic vector after excluding its *i*th item.

It is straightforward to show that $\pi |\cdots ∼\text{beta}(c_{+}+\frac{1}{2},K-c_{+}+\frac{1}{2})$. For the allocation variables it follows that

(13) $$P\left(\xi_{i}=k|\cdots \right)\propto \theta_{k}f_{k}\left(x_{i}\right),k=1,\ldots ,K,$$

(14) $$P\left(z_{j}=k|\cdots \right)\propto w_{k}f_{k}\left(y_{i}\right),k=1,\ldots ,K$$

independent for *i*=1,…,*r* and *j*=1,…,*s*. Now, given (**u**,**v**,*c*,*τ*), it is again trivial to see that the full conditional distribution of ***θ***,**w**|⋯ is the same as in equation (7). Let GD(·,·) denote the generalized Dirichlet distribution (see appendix B in the on‐line supplementary material) and also define

$$\begin{array}{c} s_{k}\left(\xi \right):=\sum_{i=1}^{r}I\left(\xi_{i}=k\right),\\ s_{k}\left(z\right):=\sum_{j=1}^{s}I\left(z_{j}=k\right) \end{array}$$

for *k*=1,…,*K*. Regarding the full conditional distribution of the free parameters, we have the following result.

Lemma 2 The full conditional distribution of (**u**,**v**|⋯) is

(15) $$u|\cdots ∼\text{GD}(\lambda_{1},\ldots ,\lambda_{K-1};\beta_{1},\ldots ,\beta_{K-1}),$$

(16) $$v|\cdots ∼D_{c_{+}-1}\left[\left\{\gamma_{l}+s_{\tau_{l+k^{*}}}\left(z\right);l=1,\ldots ,c_{+}\right\}\right],$$

with $k^{*}:=K-c_{+}$, conditionally independent (given all other variables), where

$$\lambda_{k}:=\left\{\begin{array}{cc} \alpha_{k}+s_{\tau_{k}}\left(\xi \right)+s_{\tau_{k}}\left(z\right), & k=1,\ldots ,k^{*},\\ \alpha_{k}+s_{\tau_{k}}\left(\xi \right), & k=k^{*}+1,\ldots ,K-1. \end{array}\right.$$

and

$$\beta_{k}:=\left\{\begin{array}{cc} \sum_{j=k+1}^{K}\{\alpha_{j}+s_{\tau_{j}}\left(\xi \right)+s_{\tau_{j}}\left(z\right)\}, & k=1,\ldots ,k^{*},\\ \sum_{j=k+1}^{K}\{\alpha_{j}+s_{\tau_{k}}\left(\xi \right)\}, & k=k^{*}+1,\ldots ,K-1. \end{array}\right.$$

For a proof of lemma 2, see appendix D in the on‐line supplementary material.

Here, we underline that we have essentially derived an alternative construction of the generalized Dirichlet distribution. Assuming that two vectors of weights share some common elements, and independent Dirichlet prior distributions are assigned to the free parameters of these weights, the posterior distribution of the first free‐parameter vector is a generalized Dirichlet distribution. Finally, note that, if **v**=∅ (this is the case when the corresponding elements of the weights of the two mixtures are all equal to each other), the generalized Dirichlet distribution (15) reduces to the distribution $D_{K-1}\left[\left\{\alpha_{k}+s_{k}\left(\xi \right)+s_{k}\left(z\right);k=1,\ldots ,K\right\}\right]$, as expected, since in such a case (**x**,**y**) forms a random sample of size *r*+*s* from the same population. However, if all weights are different, the full conditional distribution of **u** and **v** becomes a product of two independent Dirichlet distributions, as expected. Next we show that we can integrate out the parameters that are related to transcript expression and directly sample from the marginal posterior distribution of ***ξ***,**z**,*c*|**x**,**y**.

Theorem 2 Integrating out the transcript expression parameters **u** and **v**, the full conditional distributions of allocation variables are written as

(17) $$\begin{array}{cc} f(\xi ,z|x,y,c) & \propto \frac{\Gamma \left\{\sum_{k\in C_{1}}{\tilde{\alpha }}_{k}+s_{k}\left(\xi \right)+s_{k}\left(z\right)\right\}}{\Gamma \left\{\sum_{k\in C_{1}}{\tilde{\alpha }}_{k}+s_{k}\left(\xi \right)\right\}\Gamma \left\{\sum_{k\in C_{1}}\gamma_{l(k)}+s_{k}\left(z\right)\right\}}\\ & \;\times \prod_{k\in C_{1}}\Gamma \left\{{\tilde{\alpha }}_{k}+s_{k}\left(\xi \right)\right\}\Gamma \left\{\gamma_{l(k)}+s_{k}\left(z\right)\right\}\\ & \;\times \prod_{k\in C_{0}}\Gamma \left\{{\tilde{\alpha }}_{k}+s_{k}\left(\xi \right)+s_{k}\left(z\right)\right\}\prod_{i=1}^{r}f_{\xi_{i}}\left(x_{i}\right)\prod_{j=1}^{s}f_{z_{j}}\left(y_{j}\right) \end{array}\;,$$

(18) $$P\left(\xi_{i}=k|\xi_{\left[-i\right]},z,c,x\right)\propto \left\{\begin{array}{cc} \left\{{\tilde{\alpha }}_{k}+s_{k}^{\left(i\right)}\left(\xi \right)+s_{k}\left(z\right)\right\}f_{k}\left(x_{i}\right), & k\in C_{0},\\ \frac{\sum_{t\in C_{1}}{\tilde{\alpha }}_{k}+s_{t}^{\left(i\right)}\left(\xi \right)+s_{t}\left(z\right)}{\sum_{t\in C_{1}}{\tilde{\alpha }}_{t}+s_{t}^{\left(i\right)}\left(\xi \right)}\left\{{\tilde{\alpha }}_{k}+s_{k}^{\left(i\right)}\left(\xi \right)\right\}f_{k}\left(x_{i}\right), & k\in C_{1}, \end{array}\right.$$

(19) $$P\left(z_{j}=k|z_{\left[-j\right]},\xi ,c,y\right)\propto \left\{\begin{array}{cc} \left\{{\tilde{\alpha }}_{k}+s_{k}\left(\xi \right)+s_{k}^{\left(j\right)}\left(z\right)\right\}f_{k}\left(y_{j}\right), & k\in C_{0},\\ \frac{\sum_{t\in C_{1}}{\tilde{\alpha }}_{t}+s_{t}\left(\xi \right)+s_{t}^{\left(j\right)}\left(z\right)}{\sum_{t\in C_{1}}\gamma_{l(t)}+s_{t}^{\left(j\right)}\left(z\right)}\left\{\gamma_{l(k)}+s_{k}^{\left(j\right)}\left(z\right)\right\}f_{k}\left(y_{j}\right), & k\in C_{1}, \end{array}\right.$$

where ${\tilde{\alpha }}_{k}=\alpha_{\tau_{k}^{-1}}$, $l\left(k\right)=\tau_{k}^{-1}-k^{*}$, $s_{k}^{\left(i\right)}\left(\xi \right)=\Sigma_{t\neq i}I\left(\xi_{i}=k\right)$ and $s_{k}^{\left(j\right)}\left(z\right)=\Sigma_{t\neq j}I\left(z_{i}=k\right)$ for *k*=1,…,*K*,* i*=1,…,*r* and *j*=1,…,*s*.

For a proof of theorem 2, see appendix E in the on‐line supplementary material.

Once again, note the intuitive interpretation of our model in the special cases where *C*_0_=∅ or *C*_1_=∅. If *C*_0_=∅ (all transcripts are differentially expressed) then the denominator in the first line of equation (17) becomes equal to Γ(Σ_*k*_ *α*_*k*_+*r*+*s*), i.e. independent of ***ξ*** and **z**. Hence, equation (17) reduces to the conditional distribution of the allocation variables when independent Dirichlet prior distributions are imposed on the mixture weights. In contrast, when *C*_1_=∅ (all transcripts are equally expressed), the distribution reduces to the product appearing in the last row of equation (17). This is the marginal distribution of the allocations when considering that (**x**,**y**) arise from the same population and after imposing a Dirichlet prior on the weights, as expected.

### 2.4 Markov chain Monte Carlo samplers

In this section we consider the problem of sampling from the posterior distribution of model (12). We propose two (alternative) MCMC sampling schemes, depending on whether the transdimensional random variable **v** is updated before or after *c*.

Given *c* everything has fixed dimension. However, as *c* varies on the set of its possible values, then $v\in \cup_{k\in \{0,\;2,\;\ldots \;\;\ldots ,\;K\}}P_{k-1}$. This means that, whenever *c* is updated, **v** should change dimension. To construct a sampler that switches between different dimensions, an RJMCMC method (Green, 1995) can be implemented (see also Richardson and Green (1997) and Papastamoulis and Iliopoulos (2009)). However, this step can be avoided since we have already shown that the transcript expression parameters can be integrated out. Thus, a collapsed sampler is also available. Given an initial state, the general workflow for the samplers proposed is shown in Table 1 (we avoid explicitly stating that all distributions appearing in Table 1 are conditionally defined on the observed data **x** and **y**, although they should be understood as such).

**Table 1:** Workflow for the two samplers

*RJMCMC sampler*	*Collapsed sampler*
(a) Update (***ξ***,**z**)|***θ***,**w**	(a) Update $\xi_{i}|\xi_{\left[-i\right]},z,c$, *i*=1,…,*r*
(b) Update (**u**,**v**)|*c*,***ξ***,**z**	(b) Update $z_{j}|\xi ,z_{\left[-j\right]},c$, *j*=1,…,*s*
(c) Update (***θ***,**w**)|*c*,*τ*,**u**,**v**	(c) Update a block of *c*|***ξ***,**z**
(d) Propose update of (*c*,*τ*,**v**)|…	(d) Update *π*|*c*
(e) Update *π*|*c*	(e) Update (***θ***,**w**,*τ*,**u**,**v**)|*c*,***ξ***,**z** (optional)

Note that step (e) is optional for the collapsed sampler. It is implemented only to derive the estimates of transcript expression but it is not necessary for the previous steps. The next paragraphs outline the workflow for step (d) of the RJMCMC sampler and step (c) of the collapsed sampler. For full details the reader is referred to appendices F and G in the on‐line supplementary material.

- *Reversible jump sampler*: models of different dimensions are bridged by using two move types, namely ‘birth’ and ‘death’ of an index. The effect of a birth or death move is respectively to increase or decrease the number of differentially expressed transcripts. These moves are complementary in the sense that the one is the reverse of the other. Note that this step proposes a candidate state which is accepted according to the acceptance probability.
- *Collapsed sampler*: in this case we randomly choose two transcripts (*j*_1_ and *j*_2_) and perform an update from the conditional distribution $c_{j_{1},\;j_{2}}|c_{-[j_{1},\;j_{2}]}\xi ,z,x,y,\pi$, which is detailed in equations (G.1)–(G.4) in section G of the on‐line supplementary material. The random selection of the block {*j*_1_,*j*_2_}⊆{1,…,*K*} and the corresponding update of $c_{j_{1},\;j_{2}}$ from its full conditional distribution is a valid MCMC step because it corresponds to a Metropolis–Hastings step in which the acceptance probability equals 1 (see lemma 2 in appendix G of the supplementary material).

### 2.5 Clustering of reads and transcripts

In real RNA‐seq data sets the number of transcripts could be very large. This imposes a great obstacle for the practical implementation of the approach proposed: the search space of the MCMC sampler consists of 2^*K*^ elements (state vectors) and convergence of the sampler may be very slow. This problem can be alleviated by a cluster representation of aligned reads to the transcriptome. High quality mapped reads exhibit a sparse behaviour in terms of their mapping places: each read aligns to a small number of transcripts and there are groups of reads mapping to specific groups of transcripts. Hence, we can take advantage of this sparse representation of alignments and break the initial problem into simpler problems, by performing MCMC sampling per cluster.

This clustering representation introduces an efficient way to perform parallel MCMC sampling by using multiple threads for transcript expression estimation. For this purpose we used the GNU parallel (Tange, 2011) tool, which effectively handles the problem of splitting a series of jobs (MCMC sampling per cluster) into the available threads. The jobs are ordered according to the number of reads per cluster and those containing more reads are queued first. GNU parallel efficiently spawns a new process when one finishes and keeps all available central processor units active, thus saving time compared with an arbitrary assignment of the same amount of jobs to the same number of available threads. For further details see the on‐line appendix H.

### 2.6 False discovery rate

Controlling the FDR (Benjamini and Hochberg, 1995; Storey, 2003) is a crucial issue in multiple‐comparisons problems. Under a Bayesian perspective, any probabilistic model that defines a positive prior probability for DE and expression estimation yields that $E\left(\text{FDR}|\text{data}\right)=\Sigma \left\{1-\hat{P}\left(c_{k}=1|x,y\right)\right\}d_{k}/D$ (see for example Müller *et al*. (2006, 2004)), where $d_{k}\in \left\{0,1\right\}$ and *D*=Σ *d*_*k*_ denote the decision for transcript *k*,* k*=1,…,*K*, and the total number of rejections respectively. Consequently, the FDR can be controlled at a desired level *α* by choosing the transcripts that $\hat{P}\left(c_{k}=1|x,y\right)>1-\alpha$, which is also the approach that was proposed by Leng *et al*. (2013). We have found that this rule achieves small FDRs compared with the desired level *α*, but sometimes results in a small true positive rate.

A less conservative choice is as follows. Let *q*_1_⩾  …⩾*q*_*K*_ denote the ordered values of $\hat{P}\left(c_{k}=1|x,y\right)$, *k*=1,…,*K*, and define $G_{k}:=\Sigma_{j=1}^{k}\left(1-q_{k}\right)/k$, *k*=1,…,*K*. For any given 0<*α*<1, consider the decision rule

(20) $$d_{k}=\left\{\begin{array}{cc} 1, & 1⩽k⩽g,\\ 0, & g+1⩽k⩽K. \end{array}\right.$$

where *g*:=max{*k*=1,…,*K*:*G*_*k*_⩽*α*}. It is quite straightforward to see that expression (20) controls the expected FDR at the desired level *α*, since by direct substitution we have that

$$E\left(\text{FDR}|\text{data}\right)=\frac{\sum_{k=1}^{K}\left\{1-\hat{P}\left(c_{k}=1|x,y\right)\right\}d_{k}}{D}=\frac{\sum_{k=1}^{g}\left(1-q_{k}\right)}{g}⩽\alpha .$$

An alternative is to use a rule optimizing the posterior expected loss of a predefined loss function. For example, the threshold *c*/(*c*+1) is the optimal cut‐off under the loss function *L*=*c* FD+FN, where FD and FN denote the posterior expected counts of false discoveries and false negative discoveries respectively. Note that *L* is an extension of the (0,1,*c*) loss functions for traditional hypothesis testing (Lindley, 1971), whereas a variety of alternative loss functions can be devised as discussed in Müller *et al*. (2004).

## 3 Results

A set of simulation studies is used to benchmark the proposed methodology by using synthetic RNA‐seq reads from the *Drosophila melanogaster* transcriptome. The Spanki software (Sturgill *et al*., 2013) is used for this. In addition to the simulated data study we also perform a comparison for two real data sets: a low and high coverage sequencing experiment using human data and a data set from drosophila. In all cases, the reads are mapped to the reference transcriptome by using Bowtie (version 2.0.6), allowing up to 100 alignments per read. TopHat (version 2.0.9) is also used for Cufflinks.

### 3.1 Evaluation of samplers

**Figure 3:**
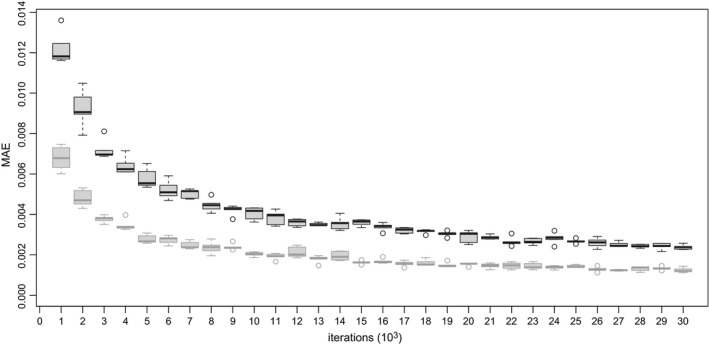
Convergence of the ergodic means of posterior probabilities of DE for a toy example of *K*= 630 transcripts (the ‘ground truth’ for the posterior mean estimates (${\hat{P}}_{g}\left(c_{k}\text{=}1\right)$; *k*=1,...,*K*) of these probabilities was inferred by running each sampler for 500000 iterations; then, each sampler ran for a smaller number of *m* iterations resulting in the posterior mean estimates ${\hat{P}}_{m}\left(c_{k}\text{=}1\right)$, *k*=1,...,*K*, for *m*=1000,2000,...,30000; finally, the averaged mean absolute error of the posterior mean estimates was computed as $\left(1/K\right)\Sigma_{k\text{=}1}^{K}\left|{\hat{P}}_{m}\left(c_{k}\text{=}1\right)-{\hat{P}}_{g}\left(c_{k}\text{=}1\right)\right|$; the boxplots correspond to five replications of the previous procedure): 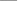, collapsed sampler; 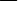, RJMCMC sampler

We used a simulated data set from *K*=630 transcripts (more details are described in the on‐line appendix H) and compare the posterior mean estimates between short and long runs. As shown in Fig. 3, the collapsed sampler exhibits faster convergence than the RJMCMC sampler; hence in what follows we shall present only results corresponding to the collapsed sampler. The reader is referred to the on‐line supplementary material (appendices J and K) for further comparisons (including auto‐correlation function estimation and prior sensitivity) between our two MCMC schemes.

### 3.2 Simulated data

The input of the Spanki simulator is a set of reads per kilobase values per sample. This file is provided under a variety of generative scenarios. Given the input files, Spanki simulates RNA‐seq reads (in ‘fastq’ format, a text‐based format for storing nucleotide sequences with the corresponding quality scores) according to the specified reads per kilobase values. Seven scenarios are used to generate the data: two Poisson replicates per condition (scenario 1), three negative binomial replicates per condition (scenario 2), nine negative binomial replicates (scenario 3), three negative binomial replicates per condition with five times higher variability among replicates compared with scenario 2 (scenario 4) and the same variability as scenario 4 but a smaller range for the mean reads per kilobase values (scenario 5). The last two scenarios are revisions of the first scenario with smaller fold changes (scenario 6) and large differences in the number of reads between conditions (scenario 7). See the on‐line supplementary Fig. 9 and appendix K for the details of the ground truth that was used in our simulations.

Next, we applied the method proposed and compared our results against Bitseq, Cuffdiff and EBSeq, using

**Figure 4:**
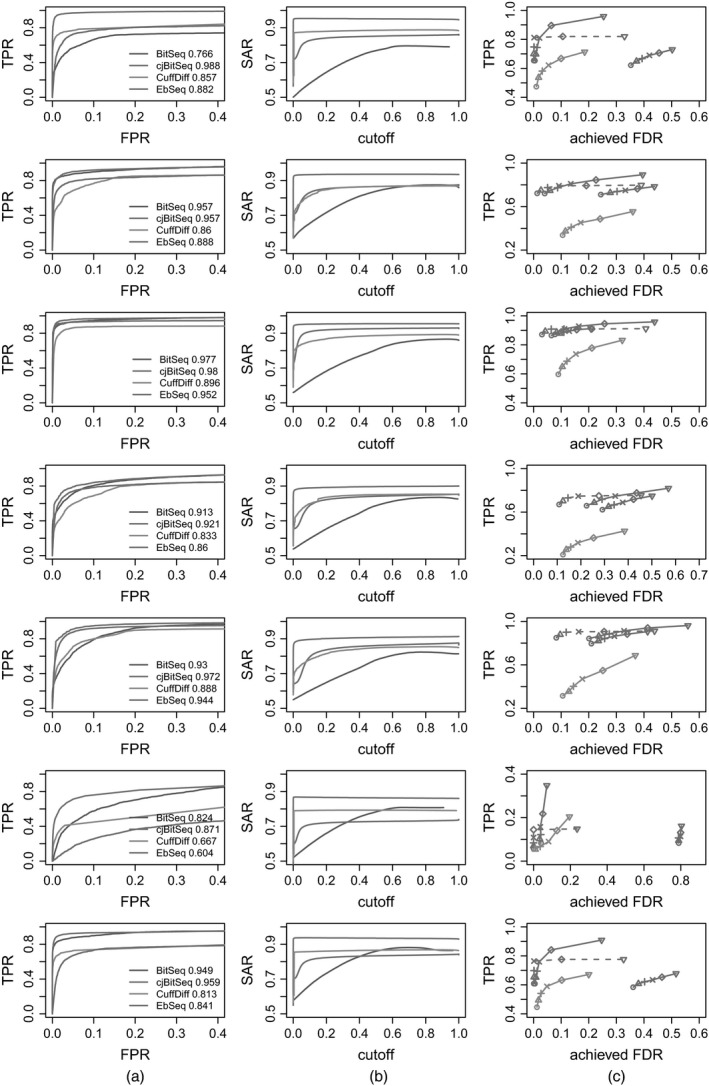
(a) Receiver operating characteristic, (b) SAR‐measure and (c) power to achieved FDR curves for scenarios 1–7 (from top to bottom): 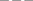, filtered cjBitSeq output by discarding transcripts with absolute log_2_‐fold change less than 1; 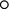, eFDR=0.01; 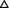, eFDR=0.025; 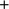, eFDR=0.05; 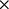, eFDR=0.1; 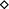, eFDR=0.2; 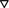, eFDR=0.4

- the receiver operating characteristic,
- the squared error, accuracy receiver operating characteristic area measure, SAR (Sing *et al*., 2005), and
- the power to achieved FDR curves, as shown in Fig. 4.

**Figure 5:**
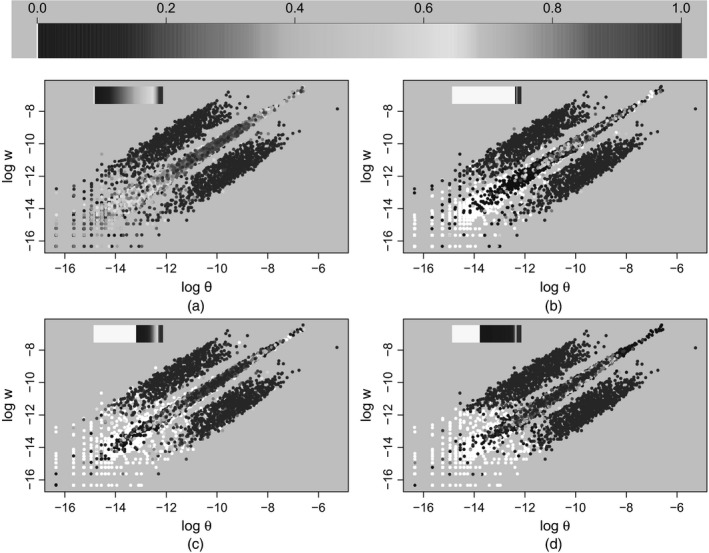
True log‐relative expression values for scenario 3 (average of nine replicates per condition: approximately 24 million reads in total) (the colour corresponds to the evidence of DE according to each method and the keys show the relative frequency of colours): (a) BitSeq; (b) cjBitSeq; (c) Cuffdiff; (d) EbSeq

For the comparison in (c) the FDR decision of our model is based on rule (20). Moreover, only methods that control the FDR are taken into account in (c); hence BitSeq stage 2 is excluded. In addition to this FDR control procedure, we also provide adjusted rates after imposing a threshold to the log‐fold change of the cjBitSeq sampler: all transcripts with estimated absolute log_2_‐fold change less than 1 are filtered out (results correspond to the broken lines in Fig. 4). A typical behaviour of the methods compared is illustrated in Fig. 5, displaying true expression values used in scenario 3. We conclude that our method infers an almost ideal classification, which is not something that applies to the other methods despite the large number of replicates used.

**Figure 6:**
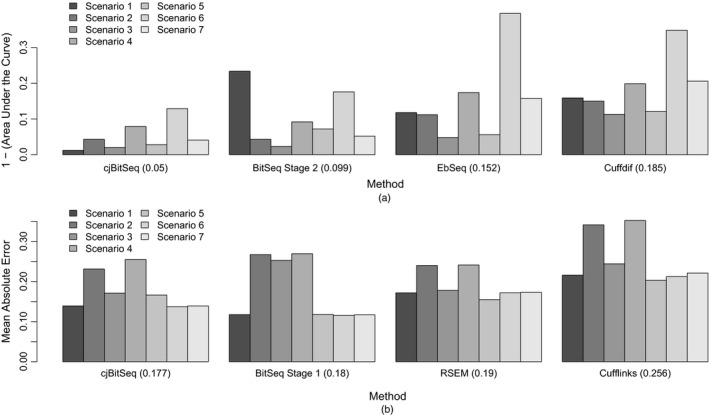
Simulated data: ranking of methods with respect to estimation of (a) DE and (b) the logarithm of relative expression (the methods are ordered according to the averaged complementary area under the curve and mean absolute error (shown in parentheses))

To summarize our findings, Fig. 6 displays the complementary area under the curve for each scenario. Averaging across all simulation scenarios, we conclude that our method is almost twice as good as BitSeq stage 2, three times better than EBSeq and 3.2 times better than Cuffdif. Finally, we compare the estimated relative abundance of transcripts against the true values that were used to generate the data, using the average across all replicates of a given condition. Fig. 6(b) displays the mean absolute error between the logarithm of true transcript expression and the corresponding estimates according to each method. We see that cjBitSeq, BitSeq stage 1 and RSEM exhibit similar behaviour, and all perform significantly better than Cufflinks. Although there is no consistent ordering between the first three methods, averaging across all experiments we conclude that cjBitSeq is ranked first.

We have also tested the sensitivity of our method with respect to the prior distributions of DE (3) by setting *π*=0.5 (see the on‐line supplementary Fig. 11 and the corresponding discussion in appendix K). We conclude that the prior distribution does not affect the ranking of methods either for DE or expression estimation.

### 3.3 Human data

This example demonstrates the proposed algorithm to differential analysis of lung fibroblasts in response to loss of the developmental transcription factor HOXA1; see Trapnell *et al*. (2013) for full details. There are three biological replicates in the two conditions. The experiment is carried out by using two sequencing platforms: ‘HiSeq’ and ‘MiSeq’, where MiSeq produced only 23% of the number of reads in the HiSeq data. Here, these reads are mapped to hg19 (University of California, Santa Cruz, gerome browser annotation) using Bowtie 2, consisting of *K*=48 009 transcripts. In total, there are 96 969 106 and 21 271 542 mapped reads for HiSeq and MiSeq sequencers respectively. Trapnell *et al*. (2013) demonstrated the ability of Cuffdiff2 to recover the transcript dynamics from the HOXA1 knockdown when using the significantly smaller amount of data generated by MiSeq compared with HiSeq.

**Figure 7:**
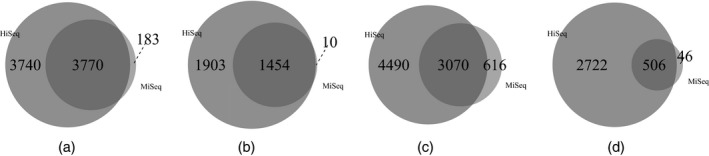
HOXA1 knockdown data set: significant transcript list returned by (a) cjBitSeq (50.2%), (b) BitSeq (43.3%), (c) EBSeq (40.6%) and (d) Cuffdiff (15.7%) when using HiSeq (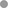) and MiSeq (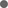) data (the FDR for cjBitSeq, EBSeq and Cuffdiff were set to 0.05, whereas, for BitSeq, PPLR<0.025 or PPLR>0.975)

**Figure 8:**
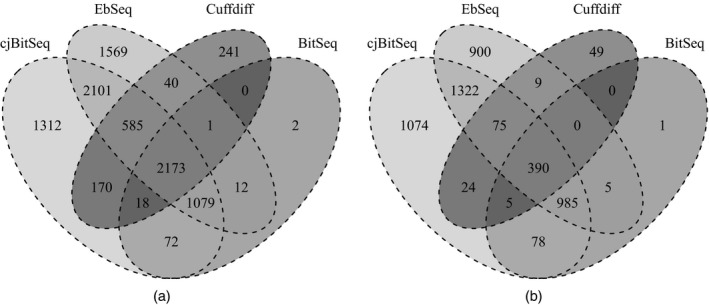
HOXA1 knockdown data set: contiguity of methods when using (a) HiSeq and (b) MiSeq data (the FDR for cjBitSeq, EBSeq and Cuffdiff were set to 0.05, whereas, for BitSeq, PPLR<0.025 or PPLR>0.975)

Applying cjBitSeq to the MiSeq data recovers 50.2% of the DE transcripts from HiSeq. In contrast, 183 transcripts are reported as differentially expressed with the MiSeq data but not the HiSeq data (Figs 7(a) and 7(b)). The corresponding percentages for BitSeq stage 2, EBSeq and Cuffdiff are 43.3%, 40.6% and 15.7% respectively (see Figs 7(b), 7(c) and 7(d)). We conclude that the model proposed returns the largest proportion of consistently differentially expressed transcripts between platforms. The number of transcripts which are simultaneously reported as differentially expressed is equal to 2173 and 390 for HiSeq and MiSeq data respectively (Figs 8(a) and 8(b)). Finally, cjBitSeq and EBSeq provide the most highly correlated classifications (see Table 1 of the on‐line supplementary material).

## 4 Discussion

We have proposed a probabilistic model for the simultaneous estimation of transcript expression and DE between conditions. Building on the BitSeq framework, the new Bayesian hierarchical model is conjugate for fixed dimension variables. A by‐product is a new interpretation of the generalized Dirichlet distribution, which naturally appears in equation (15) as the full conditional distribution of a random variable describing one of the free parameters corresponding to two proportion vectors under the constraint that some of the weights are equal to each other. We implemented two MCMC samplers, a reversible jump and a collapsed Gibbs sampler, and we found that the collapsed Gibbs sampler converged faster. To reduce the dimensionality of the parameter space greatly for inference we developed a transcript clustering approach which allows inference to be carried out independently on subsets of transcripts that share aligned reads. According to lemma 3 in the on‐line supplementary material (appendix H), this clustered version of the ordinary algorithm converges to the proper marginal distribution for each cluster. Thus, the algorithm has the nice property that it can be run in parallel for each cluster, and the memory requirements are quite low, providing a simple parallelization option.

The applications to simulated and real RNA‐seq data reveal that the method proposed is highly competitive with the current state of the art software dealing with DE analysis at the transcript level. Note that the simulated data were generated under a variety of scenarios and including different levels of replication and biological variation. We simulated transcript reads per kilobase values with variability following either the Poisson or the negative binomial distribution with various levels for the dispersion around the mean. We conclude that our method is quite robust in expression estimation and in classifying transcripts as differentially expressed or not. Compared with standard two‐stage pipelines it is ranked as the best method under a wide range of generative scenarios.

RNA‐seq data are usually replicated such that more than one data set is available for each condition. In such a way, biological variability between repetitions of the same experiment can be taken into account. The amount of variability between replicates can be quite high depending on the experimental conditions. Two‐stage approaches for estimating DE are strongly focused on modelling this interreplicate variability. This is not so for our method at present and all replicates of a given condition are effectively pooled before inference. Modelling the variability between replicates would significantly increase the complexity of our approach as it is technically challenging to retain conjugacy. However, according to our simulation studies, we have found that pooling replicates and jointly estimating expression and DE balances the loss through ignoring variability between replicates in many cases. Nevertheless, an extension also to model interreplicate variability would be very interesting and could be expected to improve performance when there is high interreplicate dispersion.

The method proposed was developed focusing on a comparison of two conditions and its extension to more general settings is another interesting area for future research. A remarkable property of the parameterization that was introduced in equations (5) and (6) is that its extension is straightforward when *J*>2: it can be shown that in this case there is one parameter of constant dimension and *J*−1 parameters of varying dimension. Let **u**=**u** ^(1)^ be the vector of relative abundances for condition 1. For a given condition *j*=2,…,*J* define a vector **v**_*j*_ containing the expression of transcripts not being equal to any of the previous conditions 1,…,*j*−1. Note that **v**_*j*_ is a random variable with varying length (between 0 and *K*). Furthermore, for *j*⩾2 define the vectors $u_{k}^{\left(j\right)}$, *k*=1,…,*j*−1, containing the expression of transcripts that are shared with condition *k* but not with 1,…,*k*−1. It follows that $u_{k}^{\left(j\right)}$ can be written as a function of **u** ^(1)^ and **v**_*k*_, *k*=1,…,*j*−1. Hence, the relative transcript expression vector for condition *j* can be expressed as a suitable permutation of $\left(u_{1}^{\left(j\right)},\ldots ,u_{j-1}^{\left(j\right)},v_{j}\right)$. However, the question of whether the model stays conjugate for fixed dimension updates remains an open problem. If yes, the design of more sophisticated move types between different models would also be crucial to the convergence of the algorithm since the search space is increased.

The source code of the proposed algorithm is compiled for Linux distributions and it is available from https://github.com/mqbssppe/cjBitSeq. The simulation pipeline is available from https://github.com/ManchesterBioinference/cjBitSeq__benchmarking. Cluster discovery and MCMC sampling are coded in R and C++ respectively. Parallel runs of the MCMC scheme are implemented by using the GNU parallel (Tange, 2011) shell tool. The computing times that are needed for our data sets are reported in the on‐line supplementary Table 2.

## 5 Supplementary material

In the on‐line supplementary material we provide the proofs of our lemmas and theorems, a detailed description of the reversible jump proposal and the Gibbs updates of the state vector of the collapsed sampler. Also included are details of alignment probabilities and some useful properties of the generalized Dirichlet distribution. We also perform various comparisons between the RJMCMC and collapsed samplers and examine their prior sensitivity. Finally we describe the generative schemes for the simulation study and some guidelines for the practical implementation of the algorithm.

## Supporting information

‘Supplementary material for the article: “A Bayesian model selection approach for identifying differentially expressed data from RNA‐seq data”’.
