# Supplementary material for: A Bayesian model selection approach for identifying differentially expressed transcripts from RNA sequencing data
Source: J R Stat Soc Ser C Appl Stat. 2017 Feb 7;67(1):3–23. doi: 10.1111/rssc.12213 (PMC5763373; doi:10.1111/rssc.12213)
Supplement: Supplementary file 1 — ‘Supplementary material for the article: “A Bayesian model selection approach for identifying differentially expressed data from RNA‐seq data”’. [file RSSC-67-3-s001.pdf]

Supplementary material for the article: "A Bayesian model selection approach for identifying differentially expressed data from RNA-seq data"

Panagiotis Papastamoulis and Magnus Rattray

September 26, 2016

Contents

A Alignment probability **1**

B The Generalized Dirichlet distribution **2**

C Proof of Theorem 1 **4**

D Proof of Lemma 2 **5**

E Proof of Theorem 2 **6**

F Update of state vector in the rjMCMC sampler **7**

G Update of state vector in the collapsed sampler **10**

H Clustering of reads and transcripts **11**

I Initialization, burn-in and number of MCMC iterations per cluster **14**

J Comparison of samplers **14**

K Simulation study details **19**

L Implementation of the algorithm **22**

M Additional tables and figures **24**

A Alignment probability

In this section the component specific density (1) is defined. For single-end reads, let $\ell_i > 0$ denotes the length of read x_i, $i = 1, \ldots, n$. Assume that x_i aligns at some position p of a given transcript k, $k = 1, \ldots, K$ and that the corresponding transcript length equals to $L_k > 0$. Note

that both L_k, ℓ_i are known quantities. The general form of observing this alignment equals to

$$f_k(x_i) = P(x_i = p|k) = \frac{b_k(p)}{\sum_{j=1}^{L_k-\ell_i+1} b_k(j)}, \tag{A.1}$$

where $b_k(j)$ denotes the bias for a particular position p on transcript k. In case of a Uniform read distribution, the previous equation reduces to:

$$f_k(x_i) = \frac{1}{L_k - \ell_i + 1}. \tag{A.2}$$

More complex choices are also available. In particular, a separate variable length Markov is used to capture the position and sequence specific biases for the $5'$ and $3'$ ends of the fragment. For more details the reader is referred to Glaus et al. (2012).

In case of paired-end reads, the fragment length ℓ is also taken into account. The fragment length distribution $f(\ell|k)$ is assumed to be log-normal with parameters given by the user or estimated from read pairs with only a single transcript alignment. In this case the alignment probability of a read pair is given as

$$f_k(x_i) = f_k(x_i = p, \ell) = f(\ell|k)\frac{b_k(p)}{\sum_{j=1}^{L_k-\ell_i+1} b_k(j)}. \tag{A.3}$$

Finally, the alignment probabilities also take into account base-calling errors using the Phred score. For full details see Glaus et al. (2012). In our presented examples we assumed the Uniform read distribution.

The sampling scheme of the RNA-seq procedure for single-end reads is displayed in Figure 1. The four long sequences of letters correspond to transcripts which share specific parts of their sequence. The gray coloured regions are skipped, so each transcript is consisting only from the remaining region (coloured in red, blue, green and purple). The short reads are randomly generated sequences from each transcript. Note that most reads align to more than one transcript.

B The Generalized Dirichlet distribution

This generalization of the Dirichlet distribution was originally introduced by Connor and Mossiman (1969). The most prominent difference with a typical Dirichlet is that the Generalized Dirichlet family has a richer covariance structure. For example, only negative correlation between any pairs of variables is allowed under the Dirichlet distribution, while the Generalized Dirichlet can also allow positive correlation. Another difference is that any permutation of a vector of proportions which follows a Dirichlet distribution is also distributed as a Dirichlet distribution. However, this is not necessarily true for the Generalized Dirichlet distribution.

In this paper we follow the parameterization of the Generalized Dirichlet distribution introduced by Wong (1998). Let $\boldsymbol{X} = (X_1, \ldots, X_k; X_{k+1})$, with $\sum_{j=1}^{k} X_j \leqslant 1$, $X_j \geqslant 0$ for $j = 1, \ldots, k$ and $X_{k+1} = 1 - X_1 - \ldots - X_k$. Assume that $\alpha_j > 0$, $\beta_j > 0$ be a set of parameters, $j = 1, \ldots, k$. Then, $\boldsymbol{X} \sim \mathcal{GD}(\alpha_1, \ldots, \alpha_k; \beta_1, \ldots, \beta_k)$ if the probability density function is written as

$$f_{\boldsymbol{X}}(\boldsymbol{x}) = \begin{cases} \prod_{j=1}^{k} \frac{x_j^{\alpha_j-1}(1-x_1-\ldots-x_j)^{\gamma_j}}{B(\alpha_j,\beta_j)}, & \sum_{j=1}^{k} x_j \leqslant 1, x_j \geqslant 0, j = 1, \ldots, k \\ 0, & \text{otherwise} \end{cases} \tag{B.1}$$

where $\gamma_j = \beta_j - \alpha_{j+1} - \beta_{j+1}$ for $j = 1, \ldots, k-1$, and $\gamma_k = \beta_k - 1$ and $B(\cdot, \cdot)$ denotes the Beta function. Note that when

$$\beta_j = \alpha_{j+1} + \beta_{j+1}, \quad j = 1, \ldots, k-1, \tag{B.2}$$

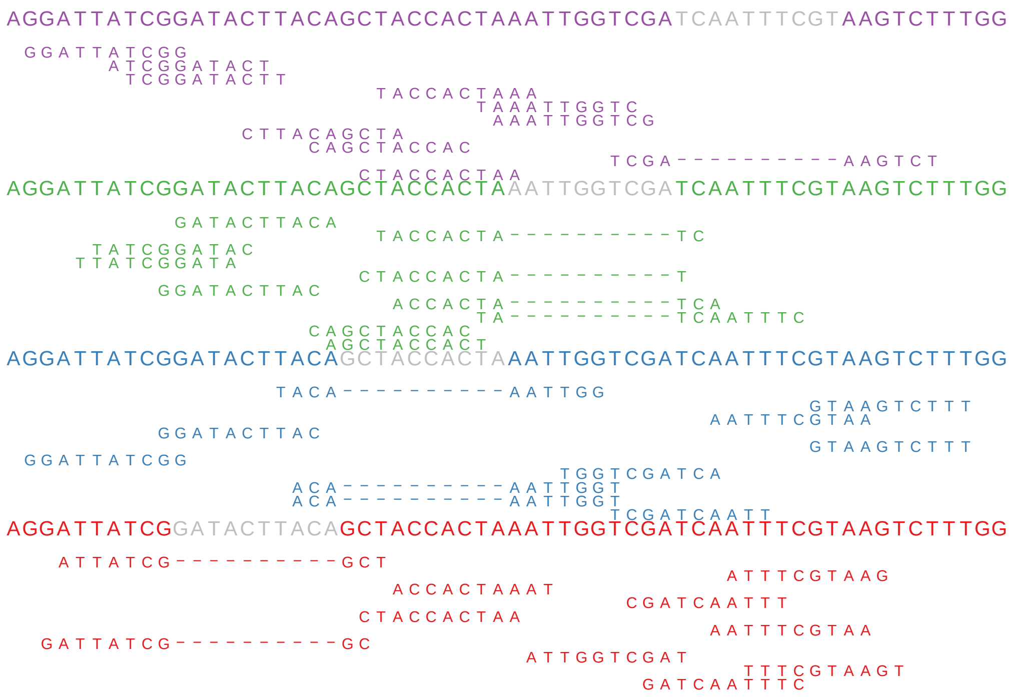

Figure 1: Illustration of the RNA-seq sampling scheme using single reads and a small set of four transcripts (red, blue, green and purple). Gray color corresponds to skipped regions (exons). From each transcript we simulated 10 reads each one consisting of 10 base pairs, displayed under each transcript.

a Generalized Distribution reduces to a standard Dirichlet distribution.

An important property of both Dirichlet and Generalized Dirichlet is that they can be constructed using a stick breaking process. The following result is from Connor and Mossiman (1969): Define $\zeta_1 = X_1$ and $\zeta_j = X_j/V_{j-1}$ for $j = 2, 3, \ldots, k$, where $V_j = 1 - X_1 - \ldots - X_{j-1}$. If $\zeta_j \sim \text{Beta}(\alpha_j, \beta_j)$, independent for $j = 1, \ldots, k$. Hence we can construct X as follows:

$$\begin{aligned} X_1 &= \zeta_1 \\ X_j &= \zeta_j(1 - X_1 - \ldots - X_{j-1}) = \zeta_j \prod_{i=1}^{j-1}(1-\zeta_i), j = 2, 3, \ldots, k \\ X_{k+1} &= 1 - \prod_{i=1}^{k}(1-\zeta_i). \end{aligned}$$

In this case: $\boldsymbol{X} = (X_1, \ldots, X_k; X_{k+1}) \sim \mathcal{GD}(\alpha_1, \ldots, \alpha_k; \beta_1, \ldots, \beta_k)$ (Connor and Mossiman, 1969). Notice that if $\beta_j = \sum_{k=j+1}^{k+1} \alpha_k$ and also define $\beta_{k+1} = \alpha_{k+1}$ for a given $\alpha_{k+1} > 0$, then $\boldsymbol{X} \sim \mathcal{D}(\alpha_1, \ldots, \alpha_k, \alpha_{k+1})$.

The previously described construction is closely related to the notion of neutrality which was also introduced by Connor and Mossiman (1969): "a neutral vector of proportions do not influence the proportional division of the remaining interval among the remaining variables".

In particular: a vector of proportions is completely neutral if and only if ζ_i's are mutually independent (Theorem 2, Connor and Mossiman, 1969). The concept of complete neutrality as well as the representation through the ζ random variables characterize both the Dirichlet and Generalized Dirichlet distributions and it will be useful for the proof of Theorem 1.

C Proof of Theorem 1

We start with the derivation of the marginal distribution of $\boldsymbol{\theta}$. According to (5), for any given state vector c, $\boldsymbol{\theta}$ can be expressed as a suitable permutation of a random variable $\boldsymbol{u} \sim \mathcal{D}(\alpha_1, \ldots, \alpha_K)$. Thus, we can write that:

$$f(\boldsymbol{\theta}) = \sum_{c \in \mathcal{C}} P(c) f(\tau_c^{-1} \boldsymbol{u}). \tag{C.1}$$

Now recall that any permutation of $\boldsymbol{u}$ is also distributed according to a Dirichlet distribution and its parameters are just the corresponding permutation of the initial parameters. This means that $\tau_c^{-1}\boldsymbol{u} \sim \mathcal{D}_{K-1}(\tau_c^{-1}\boldsymbol{\alpha})$, where $\boldsymbol{\alpha} = (\alpha_1, \ldots, \alpha_K)$. Hence, in the general case where $\boldsymbol{\alpha}$ is an arbitrary vector of strictly positive numbers, (C.1) is a mixture of Dirichlet distributions. Now notice that if $\alpha_k = \alpha > 0$, for all $k = 1, \ldots, K$, then $\tau_c^{-1}\boldsymbol{\alpha} = \boldsymbol{\alpha}$ for $c \in \mathcal{C}$ and (C.1) reduces to $\mathcal{D}_{K-1}(\boldsymbol{\alpha})$.

The analogous result for $\boldsymbol{w}$ demands a little bit more effort. At first notice that for any given c, $\boldsymbol{w}$ can be expressed according to Equation (6) as a suitable permutation of

$$\boldsymbol{\rho} = (u_1, \ldots, u_{k^*}, v_1 D_c, \ldots, v_{c_+} D_c),$$

where $D_c = \sum_{k=k^*+1}^K u_k$. Following the similar argument with $\boldsymbol{\theta}$, it will be sufficient to prove that $\boldsymbol{\rho}$ follows a Dirichlet distribution. From the discussion in Appendix B, it is equivalent to establish that $\boldsymbol{\rho}$ is completely neutral with $\zeta_k \sim \text{Beta}(\delta_j, \sum_{k=j+1}^K \delta_k)$ independent for $k = 1, \ldots, K-1$ for some $\delta_k > 0$, $k = 1, \ldots, K$.

Let us define now the following variables:

$$\begin{aligned}
\zeta_1 &= u_1 \\
\zeta_2 &= \frac{u_2}{1-u_1} \\
&\vdots \\
\zeta_{k^*} &= \frac{u_{k^*}}{1-u_1-\ldots-u_{k^*-1}} \\
\zeta_{k^*+1} &= v_1 \\
\zeta_{k^*+2} &= \frac{v_2}{1-v1} \\
&\vdots \\
\zeta_{K-1} &= \frac{v_{K-1}}{1-v_1-\ldots-v_{K-2}}.
\end{aligned}$$

Since $\boldsymbol{u}$ and $\boldsymbol{v}$ are independent and distributed according to (9) and (10) it follows that $\zeta_k \sim \text{Beta}(\alpha_k, \sum_{j=k+1}^K \alpha_j)$ for $k = 1, \ldots, k^*$ and $\zeta_{k^*+\ell} \sim \text{Beta}(\gamma_\ell, \sum_{j=\ell+1}^{c_+} \gamma_j)$ for $\ell = 1, \ldots, c_+$. Furthermore, ζ_k are mutually independent for $k = 1, \ldots, K-1$.

Now, observe that $\rho_1 = \zeta_1$, $\rho_k = \frac{\zeta_k}{1-\rho_1-\ldots-\rho_{k-1}}$, $k = 2, \ldots, K-1$ and $\rho_K = 1 - \sum_{j=1}^{K-1} \rho_j$. But ζ's are mutually independent and Beta distributed, consequently ρ follows a Generalized

Dirichlet distribution:

$$\boldsymbol{\rho} \sim \mathcal{GD}\left(\alpha_1, \ldots, \alpha_{k^*}, \gamma_1, \ldots, \gamma_{c_+}; \sum_{j=2}^{K} \alpha_j, \ldots, \sum_{j=k^*+1}^{K} \alpha_j, \sum_{j=2}^{c_+} \gamma_j, \ldots, \gamma_{c_+}\right). \tag{C.2}$$

Since $\boldsymbol{w} = \tau_c^{-1}\rho$ for any given c, in general, the marginal prior distribution of $\boldsymbol{w}$ is a mixture of permutations of Generalized Dirichlet distributions (as previously discussed, the Generalized Dirichlet distribution is not permutation invariant). In the special case that $\alpha_k = \gamma_k = \alpha > 0$ for all $k = 1, \ldots, K$, the property (B.2) implies that the distribution (C.2) reduces to $\mathcal{D}(\boldsymbol{\alpha})$. The result follows using the same argument as the one used for $\boldsymbol{\theta}$.

D Proof of Lemma 2

From (12) we have that:

$$\begin{aligned}
\boldsymbol{u}, \boldsymbol{v} | \cdots \propto & \prod_{i=1}^{r} \boldsymbol{\theta}(\tau, \boldsymbol{u})_{\xi_i} \prod_{j=1}^{s} \boldsymbol{w}(\tau, \boldsymbol{u}, \boldsymbol{v})_{z_j} \prod_{k=1}^{K} u_k^{\alpha_k - 1} \prod_{\ell=1}^{c+} v_\ell^{\gamma_\ell - 1} \\
\propto & \prod_{k=1}^{K} \boldsymbol{\theta}(\tau, \boldsymbol{u})_k^{s_k(\boldsymbol{\xi})} \prod_{k=1}^{K} \boldsymbol{w}(\tau, \boldsymbol{u}, \boldsymbol{v})_k^{s_k(\boldsymbol{z})} \prod_{k=1}^{K} u_k^{\alpha_k - 1} \prod_{\ell=1}^{c+} v_\ell^{\gamma_\ell - 1} \\
\propto & \prod_{k=1}^{K} \tau^{-1} \boldsymbol{u}_k^{s_k(\boldsymbol{\xi})} \prod_{k \in C_0(c)} \boldsymbol{w}(\tau, \boldsymbol{u})_k^{s_k(\boldsymbol{z})} \prod_{k \in C_1(c)} \boldsymbol{w}(\tau, \boldsymbol{u}, \boldsymbol{v})_k^{s_k(\boldsymbol{z})} \\
& \times \prod_{k=1}^{K} u_k^{\alpha_k - 1} \prod_{\ell=1}^{c+} v_\ell^{\gamma_\ell - 1} \\
\propto & \prod_{k=1}^{K} u_k^{s_{\tau_k}(\boldsymbol{\xi})} \prod_{k=1}^{k^*} u_k^{s_{\tau_k}(\boldsymbol{z})} \prod_{k=k^*+1}^{K} \left(v_{k-k^*} \sum_{j=k^*+1}^{K} u_j \right)^{s_{\tau_k}(\boldsymbol{z})} \\
& \times \prod_{k=1}^{K} u_k^{\alpha_k - 1} \prod_{\ell=1}^{c_+} v_\ell^{\gamma_\ell - 1} \\
\propto & \prod_{k=1}^{k^*} u_k^{\alpha_k + s_{\tau_k}(\boldsymbol{\xi}) + s_{\tau_k}(\boldsymbol{z}) - 1} \prod_{k=k^*+1}^{K} u_k^{\alpha_k + s_{\tau_k}(\boldsymbol{\xi}) - 1} \left(\sum_{j=k^*+1}^{K} u_j \right)^{\sum_{j=k^*+1}^{K} s_{\tau_j}(\boldsymbol{z})} \\
& \times \prod_{\ell=1}^{c_+} v_\ell^{\gamma_\ell + s_{\tau_{\ell+k^*}}(\boldsymbol{z}) - 1}
\end{aligned} \tag{D.1}$$

The last expression yields to conditional independence of $\boldsymbol{u}$ and $\boldsymbol{v}$. Moreover, it is straightforward to see that the full conditional distribution of $\boldsymbol{v}$ is the one defined in expression (16). The easiest way to see that the conditional distribution of $\boldsymbol{u}$ is the one defined in (15) is to evaluate the density function (B.1) with the parameters given in (15), make all simplifications and then end up to the first row of last equation.

Finally, it is important to stress here the convenience of defining $\boldsymbol{u}$ in a way that the set of Equally Expressed transcripts (C_0) is followed by the set of Differentially Expressed transcripts

(C_1), as well as the permutation of the indices as in Definition 2. Note that the term corresponding to $\sum_{j=k^*+1}^{K} u_j$ in expression D.1 refers to the sum of weights of the Differentially Expressed transcripts. If C_1 would be a random subset of indices and not the one corresponding to the last $c_+ = K - k^*$ ones, then it would not be possible to directly express the first line of D.1 as a member of the Generalized Dirichlet family, but rather as a permutation of a Generalized Dirichlet distributed random variable.

E Proof of Theorem 2

Let $\mathcal{A}_c = \mathcal{P}_{K-1} \times \mathcal{P}_{c_+-1}$ and also note that when $c_+ = 0$ then $\mathcal{A}_c$ reduces to $\mathcal{P}_{K-1}$. From Equation (12) and Lemma 2 we have that:

$$f(\boldsymbol{\xi}, \boldsymbol{z}|\boldsymbol{x}, \boldsymbol{y}, c) \propto \int_{\mathcal{A}_c} H(\boldsymbol{u}, \boldsymbol{v}, \boldsymbol{\xi}, \boldsymbol{z}, c)\mathrm{d}\boldsymbol{u}\mathrm{d}\boldsymbol{v} \prod_{i=1}^{r} f_{\xi_i}(x_i) \prod_{j=1}^{s} f_{z_j}(y_j), \tag{E.1}$$

where $H(\boldsymbol{u}, \boldsymbol{v}, \boldsymbol{\xi}, \boldsymbol{z}, c)$ denotes the expression (D.1). Now recall that according to Lemma 2, the full conditional distribution of $\boldsymbol{u}, \boldsymbol{v}|\ldots$ becomes a product of independent Generalized Dirichlet and Dirichlet distributions. This means that

$$\begin{aligned}
\int_{\mathcal{A}_c} H(\boldsymbol{u}, \boldsymbol{v}, \boldsymbol{\xi}, \boldsymbol{z}, c)\mathrm{d}\boldsymbol{u}\mathrm{d}\boldsymbol{v} &= \prod_{k=1}^{K-1} B(\lambda_k, \beta_k) \frac{\prod_{\ell=1}^{c_+} \Gamma(\gamma_\ell + s_{\tau_{\ell+k^*}}(\boldsymbol{z}))}{\Gamma(\sum_{\ell=1}^{c_+} \gamma_\ell + s_{\tau_{\ell+k^*}}(\boldsymbol{z}))} \\
&= \prod_{k=1}^{K-1} B(\lambda_k, \beta_k) \frac{\prod_{k\in C_1} \Gamma(\gamma_{\tau_k^{-1}-k^*} + s_k(\boldsymbol{z}))}{\Gamma(\sum_{k\in C_1} \gamma_{\tau_k^{-1}-k^*} + s_k(\boldsymbol{z}))}.
\end{aligned} \tag{E.2}$$

Define $\beta_0 = \sum_{j=1}^{K} \alpha_j + r + s$. Observe that $\beta_k + \lambda_k = \beta_{k-1}$ for all $k \neq k^* + 1$. Now simplify the product of Beta functions as follows:

$$\begin{aligned}
\prod_{k=1}^{K-1} B(\lambda_k, \beta_k) &= \prod_{k=1}^{K-1} \frac{\Gamma(\lambda_k)\Gamma(\beta_k)}{\Gamma(\lambda_k + \beta_k)} \\
&= \frac{\left(\prod_{k=1}^{k^*} \Gamma(\lambda_k)\right) \Gamma(\beta_{k^*})}{\Gamma(\beta_0)} \frac{\left(\prod_{k=k^*+1}^{K-1} \Gamma(\lambda_k)\right) \Gamma(\beta_{K-1})}{\Gamma(\beta_{k^*+1} + \lambda_{k^*+1})} \\
&= \frac{\Gamma(\beta_{k^*})\Gamma(\beta_{K-1})}{\Gamma(\beta_0)\Gamma(\beta_{k^*+1} + \lambda_{k^*+1})} \prod_{k=1}^{K-1} \Gamma(\lambda_k).
\end{aligned}$$

Substituting λ_k and β_k, $k = 1, \ldots, K-1$, the last expression yields:

$$\begin{aligned}
\prod_{k=1}^{K-1} B(\lambda_k, \beta_k) &= \frac{\Gamma\left(\sum\limits_{k\in C_1} \alpha_{\tau_k^{-1}} + s_k(\boldsymbol{\xi}) + s_k(\boldsymbol{z})\right)}{\Gamma(\beta_0)\Gamma\left(\sum\limits_{k\in C_1} \alpha_{\tau_k^{-1}} + s_k(\boldsymbol{\xi})\right)} \\
&\quad \times \prod_{k\in C_0} \Gamma(\alpha_{\tau_k^{-1}} + s_k(\boldsymbol{\xi}) + s_k(\boldsymbol{z})) \prod_{k\in C_1} \Gamma(\alpha_{\tau_k^{-1}} + s_k(\boldsymbol{\xi})).
\end{aligned} \tag{E.3}$$

Note here that $\Gamma(\beta_0)$ does not depend on $\boldsymbol{\xi}$ or $\boldsymbol{z}$, hence substituting Equations (E.2), (E.3) into (E.1) we obtain (17), as stated.

Next we proceed to deriving the distributions of $\xi_i|\boldsymbol{\xi}_{[-i]},\boldsymbol{z},\boldsymbol{y},c$ and $z_j|\boldsymbol{z}_{[-j]},\boldsymbol{z},\boldsymbol{y},c$, for $i=1,\ldots,r$; $j=1,\ldots,s$. Let us focus first at the probability a specific read $i=1,\ldots,r$ of the first condition being assigned to a specific transcript $k=1,\ldots,K$, given the allocations of all remaining reads $(\boldsymbol{\xi}_{[-i]},\boldsymbol{z})$ and the state vector (c). After discarding all irrelevant terms from Equation (17) we obtain that:

$$
\begin{aligned}
f(\xi_i|\boldsymbol{\xi}[-i],\boldsymbol{z},c,\boldsymbol{x}) \quad &\propto \quad \frac{\Gamma\left(\sum\limits_{t\in C_1}\alpha_{\tau_t^{-1}}+s_t(\boldsymbol{\xi})+s_t(\boldsymbol{z})\right)}{\Gamma\left(\sum\limits_{t\in C_1}\alpha_{\tau_t^{-1}}+s_t(\boldsymbol{\xi})\right)}\prod_{t\in C_1}\Gamma(\alpha_{\tau_t^{-1}}+s_t(\boldsymbol{\xi}))\\
&\times \quad \prod_{t\in C_0}\Gamma(\alpha_{\tau_t^{-1}}+s_t(\boldsymbol{\xi})+s_t(\boldsymbol{z}))f_{\xi_i}(x_i).
\end{aligned}
$$

Now, notice that: $s_t(\boldsymbol{\xi})=s_t^{(i)}(\boldsymbol{\xi})$ for $t\neq k$ while $s_k(\boldsymbol{\xi})=s_k^{(i)}(\boldsymbol{\xi})+1$ and recall that $\Gamma(x+1)=x\Gamma(x)$. Hence, the last equation simplifies to:

$$
P(\xi_i=k|\boldsymbol{\xi}[-i],\boldsymbol{z},c,\boldsymbol{x})\propto\begin{cases}(\alpha_{\tau_k^{-1}}+s_k^{(i)}(\boldsymbol{\xi})+s_k(\boldsymbol{z}))f_k(x_i), & k\in C_0\\ \frac{\sum_{t\in C_1}\alpha_{\tau_t^{-1}}+s_t^{(i)}(\boldsymbol{\xi})+s_t(\boldsymbol{z})}{\sum_{t\in C_1}\alpha_{\tau_t^{-1}}+s_t^{(i)}(\boldsymbol{\xi})}(\alpha_{\tau_k^{-1}}+s_k^{(i)}(\boldsymbol{\xi}))f_k(x_i), & k\in C_1\end{cases}
$$

which is Equation (18), as stated. Equation (19) is derived after following the similar arguments for $z_j|\boldsymbol{z}_{[-j]},\boldsymbol{\xi},\boldsymbol{y}$.

F Update of state vector in the rjMCMC sampler

In this section we introduce the reversible jump proposal for updating the state vector c and $\boldsymbol{v}$.

Birth move: Assume that the current state of the chain is

$$g:=(c,\tau,\boldsymbol{u},\boldsymbol{v},\boldsymbol{\theta},\boldsymbol{w},\boldsymbol{\xi},\boldsymbol{z}).$$

We propose to obtain a new state for the chain

$$g=(c,\tau,\boldsymbol{u},\boldsymbol{v},\boldsymbol{\theta},\boldsymbol{w},\boldsymbol{\xi},\boldsymbol{z})\rightarrow g'=(c',\tau',\boldsymbol{u}',\boldsymbol{v}',\boldsymbol{\theta}',\boldsymbol{w}',\boldsymbol{\xi}',\boldsymbol{z}'),$$

by a birth type move. This will increase the number of differentially expressed transcripts: either by one (if $c_+\geqslant 2$) or by two (if $c_+=0$). At first, we choose a move of this specific type with probability proportional to the number of elements in $C_0(c)$, that is, $K-c_+$. Then, if $c_+\geqslant 2$ we select at random an index $k_0\in C_0(c)$ which we propose to add to $C_1(c)$. If $c_+=0$ we select at random two indexes $\{k_1,k_2\}\in C_0(c)$ which we propose to move to (the previously empty) $C_1(c)$. The probability of selecting such a move type is,

$$
P_{\text{birth}}(c\rightarrow c')=\begin{cases}\frac{K-c_+}{K}\frac{1}{K-c_+}=\frac{1}{K}, & \text{if } 2\leqslant c_+\leqslant K-1\\ \frac{K-0}{K}\frac{1}{\binom{K}{2}}=\frac{2}{K(K-1)}, & \text{if } c_+=0.\end{cases} \tag{F.1}
$$

Moreover, define the corresponding death probability

$$P_{\text{death}}(c \to c') = \begin{cases} \frac{c_+}{K}\frac{1}{c_+} = \frac{1}{K}, & \text{if } 3 \leqslant c_+ \leqslant K \\ \frac{2}{K}, & \text{if } c_+ = 2. \end{cases} \tag{F.2}$$

If $c_+ = 0$, assume without loss of generality that $k_1 < k_2$. Then, $C_1(c') = \{k_1, k_2\}$ and $C_0(c') = \{1, \ldots, K\} - C_1(c')$. Now assume that $c_+ \geqslant 2$. It is obvious that in this case $c'_k = c_k$ for all $k \neq k_0$ and $c'_{k_0} = 1$. Moreover, the dead and alive subsets of the new state is obtained by deleting k_0 from $C_0(c)$ and adding it to $C_1(c)$. Let

$$j := \sum_{k \in C_1(c)} I(k < k_0) + 1 = \sum_{k=1}^{k_0} c_k + 1.$$

Then, the alive subset of the new state is

$$C_1(c') = \begin{cases} \{C_1(c)\}_k, & k < j \\ k_0, & k = j \\ \{C_1(c)\}_{k-1}, & j < k \leqslant c_+ + 1 \end{cases} \tag{F.3}$$

and the dead subset will simply be $C_0(c') = \{1, \ldots, K\} - C_1(c')$. Finally, the new permutation is defined as $\tau' = (C_0(c'), C_1(c'))$.

Now, we have to propose the values of $\boldsymbol{u}', \boldsymbol{v}'$. Recall that the dimension of $\boldsymbol{u}$ is always constant, but the dimension of $\boldsymbol{v}'$ will be increased by one. We consider them separately in order to keep it as simple as possible. For $\boldsymbol{u}$ we propose to jump to a new state $\boldsymbol{u}'$ which arises deterministically as the corresponding permutation of its previous values. In order to do this we just have to match the position of each element of τ' in τ. This means that

$$\boldsymbol{u}' = (\tau^{-1}\tau')\boldsymbol{u} = \tau'[(\tau^{-1}\boldsymbol{u})]. \tag{F.4}$$

In order to construct a valid Metropolis-Hastings acceptance probability for the dimension changing move from $\boldsymbol{v}$ to $\boldsymbol{v}'$, we should take into account the dimension matching assumption of Green (1995). In our set up, this assumption states that the jump from $\boldsymbol{v} \to \boldsymbol{v}'$ should be done by producing one random variable that will bridge the dimensions, that is:

$$\boldsymbol{v}' = h(\boldsymbol{v}, \delta),$$

where δ denotes a (univariate) random variable and $h(\cdot, \cdot)$ an invertible transformation. We design this transformation following similar ideas from the standard birth and death moves of Richardson and Green (1997), Papastamoulis and Iliopoulos (2009). Let $\delta \sim f_{\text{prop}}$, where f_{prop} denotes the density function of a distribution with support $(0, 1)$. Then, the new parameter is obtained as

$$\boldsymbol{v}' = h(\boldsymbol{v}, \delta) := \begin{cases} \left(v_1(1-\delta), \ldots, v_{j-1}(1-\delta), \delta, v_{j+1}(1-\delta), \ldots, v_{c_+}(1-\delta)\right), & c_+ \geqslant 2 \\ (\delta, 1-\delta), & c_+ = 0 \end{cases} \tag{F.5}$$

Finally, we have to compute the absolute value of the Jacobian of the transformation in (F.5). Now, recall that $\boldsymbol{v}$ consists of $c_+ - 1$ independent elements, so the dimension of the Jacobian is $c_+ \times c_+$ (and not $(c_+ + 1) \times (c_+ + 1)$). Then, a routine calculation leads to:

$$|J(\delta, c)| = \begin{cases} (1-\delta)^{c_+ - 1}, & c_+ \geqslant 2 \\ 1, & c_+ = 0 \end{cases} \tag{F.6}$$

The new values of transcript expression $\boldsymbol{\theta}', \boldsymbol{w}'$ are as follows. By Equations (5) and (F.4)

$$\boldsymbol{\theta}' = \tau'^{-1}\boldsymbol{u}' = \tau'^{-1}\tau'[(\tau^{-1}\boldsymbol{u})] \Rightarrow \boldsymbol{\theta}' = \boldsymbol{\theta}, \tag{F.7}$$

and applying Equation (6):

$$\boldsymbol{w}' = \tau'^{-1}\left(\{u'_{\tau'^{-1}_k} : k \in C_0(c')\}, \boldsymbol{v}' \sum_{k \in C_1(c')} u'_{\tau'^{-1}_k}\right). \tag{F.8}$$

Finally, we propose to reallocate all observations according to the new values $\boldsymbol{\theta}'$ and $\boldsymbol{w}'$. This is simply done by using the full conditional distributions of $\boldsymbol{\xi}', \boldsymbol{z}'$. Let $P(\boldsymbol{\xi}', \boldsymbol{z}'|\boldsymbol{\theta}', \boldsymbol{w}')$ denote the probability of the allocations, according to the general form given in Equations (13) and (14). Note here that such a reallocation it is not necessary, however it is suggested because improves the acceptance rate of the proposed move.

Lemma 1. *The acceptance probability of the birth move is* $\min\{1, A(g, \delta, g')\}$*, where*

$$\begin{aligned} A(g,\delta,g') = & \frac{f(\boldsymbol{x},\boldsymbol{y},\boldsymbol{z}',\boldsymbol{\xi}'|\boldsymbol{\theta}',\boldsymbol{w}')P(\boldsymbol{\xi},\boldsymbol{z}|\boldsymbol{\theta},\boldsymbol{w})}{f(\boldsymbol{x},\boldsymbol{y},\boldsymbol{z},\boldsymbol{\xi}|\boldsymbol{\theta},\boldsymbol{w})P(\boldsymbol{\xi}',\boldsymbol{z}'|\boldsymbol{\theta}',\boldsymbol{w}')} \\ \times & \frac{P_{death}(c' \to c)P(c')f(\boldsymbol{u}',\boldsymbol{v}'|\boldsymbol{\alpha},\boldsymbol{\gamma})|J(\delta,c)|}{P_{birth}(c \to c')f_{prop}(\delta)P(c)f(\boldsymbol{u},\boldsymbol{v}|\boldsymbol{\alpha},\boldsymbol{\gamma})}. \end{aligned} \tag{F.9}$$

Proof. See the acceptance probability in Green (1995). □

Note that for the Jeffeys prior (2), (3) it holds that:

$$\frac{P(c')}{P(c)} = \begin{cases} \frac{\pi}{1-\pi}, & c_+ \geqslant 2 \\ \frac{\pi^2}{(1-\pi)^2}, & c_+ = 0. \end{cases}$$

Death move: A death proposal is the reverse move of a birth. Suppose that we propose a transition

$$g = (c, \tau, \boldsymbol{u}, \boldsymbol{v}, \boldsymbol{\theta}, \boldsymbol{w}, \boldsymbol{\xi}, \boldsymbol{z}) \to g' = (c', \tau', \boldsymbol{u}', \boldsymbol{v}', \boldsymbol{\theta}', \boldsymbol{w}', \boldsymbol{\xi}', \boldsymbol{z}'),$$

via a death move. At first we choose at random an element of the alive subset and propose to move and paste it to the dead subset (in the case that the alive subset consists of only two transcripts, then we essentially setting the alive subset to the empty set). This reduces the number of differentially expressed transcripts either by one (if $c_+ \geqslant 2$) or by two (if $c_+ = 2$).

If $c_+ = 2$ let $C_1(c) = \{k_1, k_2\}$ and $\boldsymbol{v} = (v_1, v_2)$, with $v_2 = 1 - v_1$. Then the random variable that we have to produce during the reverse move is deterministically set to $\delta = v_1$, and $\boldsymbol{v}' = \emptyset$. In any other case, assume that the chosen alive transcript index is k_1, then define

$$j := \sum_{k \in C_0(c)} I(k < k_1) + 1 = \sum_{k=1}^{k_1} c_k.$$

Then, the reverse transformation of (F.5) implies that

$$(\boldsymbol{v}', \delta) = h^{-1}(\boldsymbol{v}) := \begin{cases} \left\{\left(\frac{v_1}{1-v_j}, \ldots, \frac{v_{j-1}}{1-v_j}, \frac{v_{j+1}}{1-v_j}, \ldots, \frac{v_{c_+}}{1-v_j}\right), v_j\right\}, & c_+ \geqslant 2 \\ v_j, & c_+ = 0 \end{cases} \tag{F.10}$$

Everything works in a reverse way compared to the birth move, so the acceptance probability of a death move is then simply given by $\min\{1, A^{-1}(g', v_j, g)\}$.

G Update of state vector in the collapsed sampler

According to Equation (12), the conditional distribution of c is written as:

$$f(c|\boldsymbol{\xi}, \boldsymbol{z}, \pi, \boldsymbol{x}, \boldsymbol{y}) \propto f(\boldsymbol{\xi}, \boldsymbol{z}|c, \boldsymbol{x}, \boldsymbol{y}) f(c|\pi) h_c,$$

where $f(c|\pi)$ denotes the prior distribution of c defined in Equation (3), $f(\boldsymbol{\xi}, \boldsymbol{z}|c, \boldsymbol{x}, \boldsymbol{y})$ is defined in Equation (17) of Theorem 2 and $h_c = \frac{\Gamma(\sum_{\ell=1}^{c_+}\gamma_\ell)}{\prod_{\ell=1}^{c_+}\Gamma(\gamma_\ell)}$ corresponds to the constant term of the prior distribution for $\boldsymbol{v}$. However, in order to fully update the state vector we would have to compute this quantity for all $c \in \mathcal{C}$, and this would be time consuming.

An alternative is to update two randomly selected indices, given the configuration of remaining ones. Hence, if j_1 and j_2 denote two distinct transcript indices, then we perform a Gibbs update to $c_{j_1,j_2}|c_{-[j_1,j_2]}, \boldsymbol{\xi}, \boldsymbol{z}, \pi, \boldsymbol{x}, \boldsymbol{y}$. Let $d = \sum_{k \neq j_1, j_2} c_k$. Since $c_+ = \sum_k c_k \neq 1$, we have to differentiate the subsequent procedure between the following cases: $d = 0$, $d = 1$ and $d > 1$. If $d = 0$ then $c_{j_1,j_2} \in \{(1,1),(0,0)\}$. In case that $d = 1$ then $c_{j_1,j_2} \in \{(1,1),(1,0),(0,1)\}$. Finally, if $d > 1$ then $c_{j_1,j_2} \in \{(1,1),(0,0),(1,0),(0,1)\}$. Hence, the following full conditional distribution is derived:

$$P(c_{j_1} = 1, c_{j_2} = 1|c_{-[j_1,j_2]}, \boldsymbol{\xi}, \boldsymbol{z}, \pi, \boldsymbol{x}, \boldsymbol{y}) \propto f(\boldsymbol{\xi}, \boldsymbol{z}|c, \boldsymbol{x}, \boldsymbol{y})\pi^2 h_c \tag{G.1}$$

$$P(c_{j_1} = 0, c_{j_2} = 0|c_{-[j_1,j_2]}, \boldsymbol{\xi}, \boldsymbol{z}, \pi, \boldsymbol{x}, \boldsymbol{y}) \propto \begin{cases} f(\boldsymbol{\xi}, \boldsymbol{z}|c, \boldsymbol{x}, \boldsymbol{y})(1-\pi)^2 h_c, & d \neq 1 \\ 0, & d = 1 \end{cases} \tag{G.2}$$

$$P(c_{j_1} = 1, c_{j_2} = 0|c_{-[j_1,j_2]}, \boldsymbol{\xi}, \boldsymbol{z}, \pi, \boldsymbol{x}, \boldsymbol{y}) \propto \begin{cases} f(\boldsymbol{\xi}, \boldsymbol{z}|c, \boldsymbol{x}, \boldsymbol{y})\pi(1-\pi) h_c, & d \neq 0 \\ 0, & d = 0 \end{cases} \tag{G.3}$$

$$P(c_{j_1} = 0, c_{j_2} = 1|c_{-[j_1,j_2]}, \boldsymbol{\xi}, \boldsymbol{z}, \pi, \boldsymbol{x}, \boldsymbol{y}) \propto \begin{cases} f(\boldsymbol{\xi}, \boldsymbol{z}|c, \boldsymbol{x}, \boldsymbol{y})(1-\pi)\pi h_c, & d \neq 0 \\ 0, & d = 0. \end{cases} \tag{G.4}$$

Lemma 2. *The update of a randomly selected block of c in the collapsed sampler:*

1. *Select randomly two distinct indices $\{j_1, j_2\}$ from the set $\{1, \ldots, K\}$*
2. *Update $c_{j_1,j_2}|c_{-[j_1,j_2]}, \boldsymbol{\xi}, \boldsymbol{z}, \pi, \boldsymbol{x}, \boldsymbol{y}$ as detailed in Equations* (G.1)–(G.4)

corresponds to a Metropolis-Hastings step in which the proposed state is always accepted.

Proof. Assume that the current state of the chain is $g = (c, \boldsymbol{\xi}, \boldsymbol{z}, \pi)$ and we propose to move to state $g' = (c', \boldsymbol{\xi}, \boldsymbol{z}, \pi)$, where $c'_k = c_k$ if $k \neq j_1, j_2$ and c_{j_1,j_2} is drawn from the full conditional distribution. The proposal density in this case can be expressed as

$$P(g \to g') = \frac{1}{\binom{K}{2}} f(c'_{j_1,j_2}|c_{-[j_1,j_2]}, \boldsymbol{\xi}, \boldsymbol{z}, \pi, \boldsymbol{x}, \boldsymbol{y}) \propto \frac{1}{\binom{K}{2}} f(c', \boldsymbol{\xi}, \boldsymbol{z}, \pi|\boldsymbol{x}, \boldsymbol{y}) = \frac{1}{\binom{K}{2}} f(g'|\boldsymbol{x}, \boldsymbol{y}).$$

The probability of proposing the reverse move (from g' to g) equals to

$$P(g' \to g) = \frac{1}{\binom{K}{2}} f(c_{j_1,j_2}|c_{-[j_1,j_2]}, \boldsymbol{\xi}, \boldsymbol{z}, \pi, \boldsymbol{x}, \boldsymbol{y}) \propto \frac{1}{\binom{K}{2}} f(c, \boldsymbol{\xi}, \boldsymbol{z}, \pi|\boldsymbol{x}, \boldsymbol{y}) = \frac{1}{\binom{K}{2}} f(g|\boldsymbol{x}, \boldsymbol{y}).$$

Thus, the Metropolis-Hastings ratio for the transition $g \to g'$ is expressed as

$$\frac{f(g'|\boldsymbol{x}, \boldsymbol{y})P(g' \to g)}{f(g|\boldsymbol{x}, \boldsymbol{y})P(g \to g')} = \frac{f(g'|\boldsymbol{x}, \boldsymbol{y})\frac{1}{\binom{K}{2}}f(g|\boldsymbol{x}, \boldsymbol{y})}{f(g|\boldsymbol{x}, \boldsymbol{y})\frac{1}{\binom{K}{2}}f(g'|\boldsymbol{x}, \boldsymbol{y})} = 1.$$

□

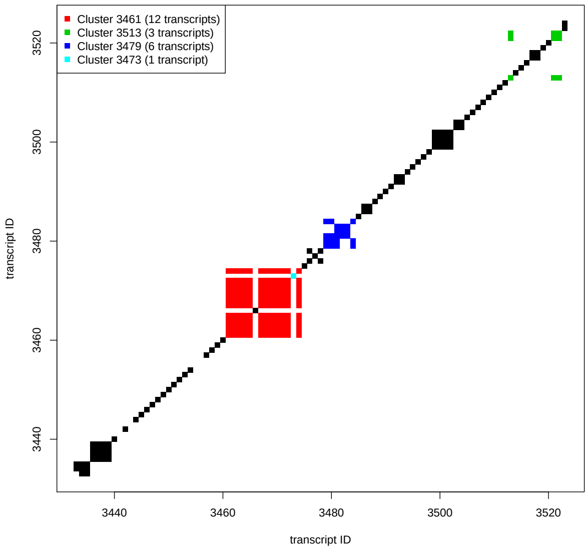

Figure 2: Clusters of transcripts for a simulated set of 75 bp paired reads from the Drosophila transcriptome, containing $K = 28763$ transcripts. For illustration purposes, only a subset consisting of 281 transcripts is shown and four clusters are emphasized using different colours. White colour corresponds to $N_{ij} = 0$ aligned reads to both transcripts i, j.

H Clustering of reads and transcripts

Let $Q = (q)_{ij}$ be a $K \times K$ symmetric matrix. For $i = 1, 2, \ldots, K$ and $j = 1, \ldots, K$ let N_{ij} denotes the number of reads that map to both transcripts i and j. Define:

$$q_{ij} := \begin{cases} 1 & \text{if } N_{ij} > 0 \\ 0 & \text{otherwise.} \end{cases}$$

Clearly, Q would be a diagonal matrix if all reads were uniquely mapped, but for real datasets Q is a sparse and almost diagonal matrix. A typical graphic representation of Q is illustrated in Figure 2 using a set of simulated reads from the Drosophila Melanogaster transcriptome. Each pixel corresponds to a pair of transcripts that contain at least one read aligned to both transcripts of the pair. If all reads were uniquely aligned, this figure would consist only of the diagonal line and in this case each expressed transcript would form its own cluster. Note that the white gaps on the diagonal line indicate non-expressed transcripts. Many reads, however, map to more than one transcript resulting in clusters of transcripts, as the red, cyan, blue and green ones in Figure 2. The number of transcripts per cluster can have a wide range of values as displayed in Figure 3, but the majority of clusters consist of a very small number of transcripts compared to their total number. Next we formally define the notion of a cluster of transcripts.

Definition 1 (Associated transcripts)**.** *Transcript k_1 is associated to transcript k_2 ($k_1 \leftrightarrow k_2$) if $q_{k_1k_2} = 1$ or if exists a subset of indices $\{i_1, i_2, \ldots, i_m\} \subseteq \mathcal{K} := \{1, \ldots, K\}$, $m \geqslant 1$, such that $q_{k_1i_1} + q_{i_1i_2} + \ldots + q_{i_{m-1}i_m} + q_{i_mk_2} = m + 1$.*

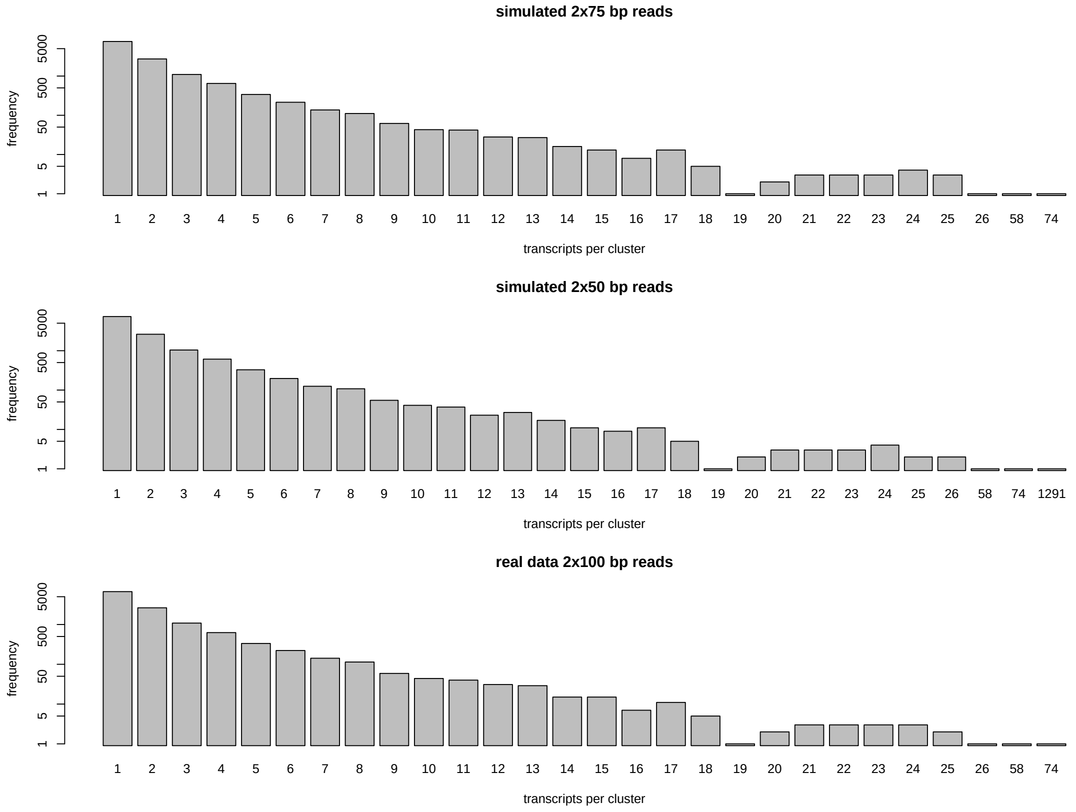

Figure 3: Frequencies (in log-scale) of the number of transcripts per cluster using paired-end reads from the Drosophila Melanogaster transcriptome. Top and middle: (4489712) simulated reads, bottom: (22571142) reads from real data.

Definition 2 (Cluster of transcripts)**.** *The set of all associated transcripts of a given transcript* $k \in \mathcal{K} : \sum_{i=1}^{K} N_{ik} > 0$*:* $\mathcal{C}_k := \{j \in \mathcal{K} : j \leftrightarrow k\}$*, denotes the cluster of* k*.*

Note that according to definition 2: $k_1 \leftrightarrow k_2 \Leftrightarrow \mathcal{C}_{k_1} = \mathcal{C}_{k_2}$. We uniquely label each cluster by referring to its minimum index, as follows:

Definition 3 (Cluster labels)**.** *The label of cluster* $\mathcal{C}_k$ *is defined as* $\mathcal{L}_\ell$*, with* $\ell = \min\{j \in \mathcal{C}_k\}$*. Conventionally, we set* $\mathcal{L}_0 := \{k \in \mathcal{K} : \mathcal{C}_k = \emptyset\}$*.*

Let n_c be the total number of clusters and assume that K_j is the number of transcripts associated with cluster $\mathcal{L}_j$. It holds that $\cup_{j=1,\ldots,n_c}\mathcal{L}_j = \mathcal{K}$ and $\mathcal{L}_i \cap \mathcal{L}_j = \emptyset$ for $i \neq j$, that is, $\{\mathcal{L}_0, \mathcal{L}_1, \ldots, \mathcal{L}_{n_c}\}$ is a partition of $\mathcal{K}$. Finally, let $r(\mathcal{L}_k)$ and $s(\mathcal{L}_k)$ be the number of reads assigned to cluster $\mathcal{L}_k$ from the first and second condition, respectively.

Next, assume that the proposed method is applied separately to each cluster. This would not lead to the same answer as the one with the full set of reads due to the fact that now each transcript weight corresponds to the relative expression inside each cluster. In order to ensure that the analysis will result to the same answer we should artificially augment each cluster with an extra pseudo-transcript that will contain information of the relative weight of each cluster. There are $r(\mathcal{L}_j)$ and $s(\mathcal{L}_j)$ reads from the first and second condition, respectively, exclusively

aligned to cluster $\mathcal{L}_j$, $j = 1, \ldots, n_c$. Equivalently, there are $r - r(\mathcal{L}_j)$ and $s - s(\mathcal{L}_j)$ reads from the first and second condition, exclusively aligning to the remaining clusters. Assume now that each cluster is augmented with an additional pseudo-transcript containing all remaining reads from both conditions. We conventionally set the label of the pseudo-transcript to $K_j + 1$. Given a set of reads from two biological conditions aligned to the reference transcriptome, the pipeline of the algorithm is the following.

- Partition the reference transcriptome and aligned reads into clusters.
- For each cluster $j = 1, \ldots, n_c$, containing $K_j \geqslant 1$ transcripts:
  - augment the cluster by the remaining pseudo-transcript containing $r - r(\mathcal{L}_j)$ and $s - s(\mathcal{L}_j)$ reads from the first and second condition, respectively.
  - Run the rjMCMC or the collapsed sampler.

The following Lemma ensures that it is valid to apply this sampling scheme per cluster in order to estimate the marginal posterior distribution of expression and differential expression for the set of transcripts assigned to each cluster. Apparently, this is not equivalent to simultaneously sampling from the joint posterior distribution of the whole transcriptome, which is computationally prohibitive, however the estimation of the marginal behaviour of each cluster is feasible and computationally efficient due to the dimension reduction.

Lemma 3. *Let $\tilde{\boldsymbol{\theta}}_j := (\{\theta_j; j \in \mathcal{L}_j\}, \sum_{k \neq \mathcal{L}_j} \theta_k)$, $\tilde{\boldsymbol{w}}_j := (\{w_j; j \in \mathcal{L}_j\}, \sum_{k \neq \mathcal{L}_j} w_k)$ denote the augmented transcript expressions for the first and second condition respectively and $\tilde{c}_j := (\{c_j; j \in \mathcal{L}_j\}, c_{K_j+1})$, at cluster $j = 1, \ldots, n_c$. A priori assume:*

$$\tilde{\boldsymbol{u}}_j \sim \mathcal{D}_{K_j}\left(\{\alpha_j; j \in \mathcal{L}_j\}, \sum_{k \notin \mathcal{L}_j} \alpha_k\right) \tag{H.1}$$

$$\tilde{\boldsymbol{v}}_j | \tilde{\boldsymbol{c}}_j \sim \mathcal{D}_{\tilde{c}_+}\left(\gamma_1, \ldots, \gamma_{K_j+1}\right). \tag{H.2}$$

Then for each cluster $j = 1, \ldots, n_c$, the parallel rjMCMC or collapsed algorithm converge to $f(\tilde{\boldsymbol{\theta}}_j, \tilde{\boldsymbol{w}}_j, \tilde{\boldsymbol{c}}_j | \boldsymbol{x}, \boldsymbol{y})$ and $f(\tilde{\boldsymbol{c}}_j | \boldsymbol{x}, \boldsymbol{y})$, respectively.

Proof. The distribution (H.1) is derived by (9) by applying the aggregation property of Dirichlet distribution, while distribution (H.2) is the same as (10) given $\boldsymbol{c} = \tilde{\boldsymbol{c}}$. Recall that according to Definition 2 there are $\sum_{i=1}^{r} I(z_i = K_j + 1) = r - r(\mathcal{L}_j)$ and $\sum_{i=1}^{s} I(\xi_i = K_j + 1) = s - s(\mathcal{L}_j)$ reads allocated to the component labelled as $K_j + 1$ for cluster j. This means that the update scheme:

1. Update allocation variables $\tilde{\boldsymbol{\xi}}_j$ and $\tilde{\boldsymbol{z}}_j$ and set $s_{K_j+1}(\tilde{\boldsymbol{\xi}}_j) := r - r(\mathcal{L}_j)$, $s_{K_j+1}(\tilde{\boldsymbol{z}}_j) := s - s(\mathcal{L}_j)$.
2. Update free parameters $\tilde{\boldsymbol{u}}_j$ and $\tilde{\boldsymbol{v}}_j$
3. Update expression parameters $\tilde{\boldsymbol{\theta}}_j$ and $\tilde{\boldsymbol{w}}_j$
4. Update state vector $\tilde{\boldsymbol{c}}_j$

updates the collapsed parameter vector:

$$\left(\{\theta_j; j \in \mathcal{L}_j\}, \sum_{k \neq \mathcal{L}_j} \theta_k\right), \left(\{w_j; j \in \mathcal{L}_j\}, \sum_{k \neq \mathcal{L}_j} w_k\right), \left(\{c_j; j \in \mathcal{L}_j\}, c_{K_j+1}\right)$$

using the full conditional distributions for steps 1, 2, 3 and the reversible jump acceptance ratio in step 4 (in the case of rjMCMC sampler) or the random scan Gibbs step (in case of collapsed Gibbs). Hence it converges to $f(\tilde{\boldsymbol{\theta}}_j, \tilde{\boldsymbol{w}}_j, \tilde{\boldsymbol{c}}_j | \boldsymbol{x}, \boldsymbol{y})$. □

Note that the previous result assumes a fixed prior probability of DE. In practice, the prior probability of DE is a random variable, following the Jeffrey's prior distribution. Hence, the clustered sampling is equivalent to joint sampling only in case of fixed prior probability of DE. But we have found that this has not any impact in practice since according to our simulations the Jeffrey's prior outperforms the fixed prior probability of DE.

If the reads are sufficiently large, the clusters of transcripts are essentially genes (or groups of genes). It should be clear that the number of clusters as well as the cluster with the largest number of transcripts depends on the read length: if the read length is small, there will be many reads that map to multiple genes and in such a case all of these reads will form a very large cluster, as the one displayed in Figure 3 (middle) containing 1291 transcripts. The convergence of the MCMC algorithm for such clusters is questionable. However, even in such cases the majority of transcripts and reads are still forming a large number of small clusters. It is worth mentioning here that the large cluster is created by a very small number of reads: in total there are 417709 reads belonging to this cluster. However, the number of reads that actually map to more than ones genes is equal to 1474. Hence, we could break the bonds of this large number of transcripts by simply discarding or filtering out this small portion of reads.

I Initialization, burn-in and number of MCMC iterations per cluster

After partitioning the reads and transcripts into clusters, the rjMCMC or collapsed sampler is applied as previously discussed. For each run (MCMC per cluster), mcmc_n independent chains are obtained using randomly selected initial values for parameters $\boldsymbol{u}$ and $\boldsymbol{v}$, drawn from (9) and (10). The first half of the chains is initialized from $c_+ = 0$ (all transcripts are equally expressed), while the reverse (all transcripts are differentially expressed) holds for the initial state of the second half. The pseudo-transcript of each cluster (i.e. the mixture component labelled as K_j+1) is always initialized as differentially expressed. Given $c, \boldsymbol{u}, \boldsymbol{v}$, the initial relative transcript expressions are computed according to (7) and (8). Each chain runs for a fixed number of mcmc_m iterations, following a pre-specified number mcmc_b of burn-in draws. The posterior means are estimated by averaging the ergodic means across all chains, using a thinning of mcmc_t steps. The proposal distribution in the reversible jump step is an equally weighted finite mixture of Beta distributions: $f_{\text{prop}} = \frac{1}{J}\sum_{j=1}^{J}\mathcal{B}(1, \beta_j)$. All results reported are obtained using: $\text{mcmc}_n = 6$, $\text{mcmc}_m = 5000$, $\text{mcmc}_b = 1000$, $\text{mcmc}_t = 5$, $J = 5$ and $\{\beta_j; j = 1, \ldots, 5\} = \{1, 10, 100, 250, 500\}$.

J Comparison of samplers

In this section we compare the Reversible Jump and the Collapsed version of our method as well we test the sensitivity of these samplers with respect to the prior probability of Differential Expression. In particular, we compare the Jeffreys' prior with a fixed prior probability of DE at 0.05, 0.50 and 0.95. We also examine the acceptance rates of the reversible jump proposal for updating the state vector c. Finally, a comparison between the clusterwise and raw sampler is made. For this purpose we used a toy example with relatively small number of reads and transcripts.

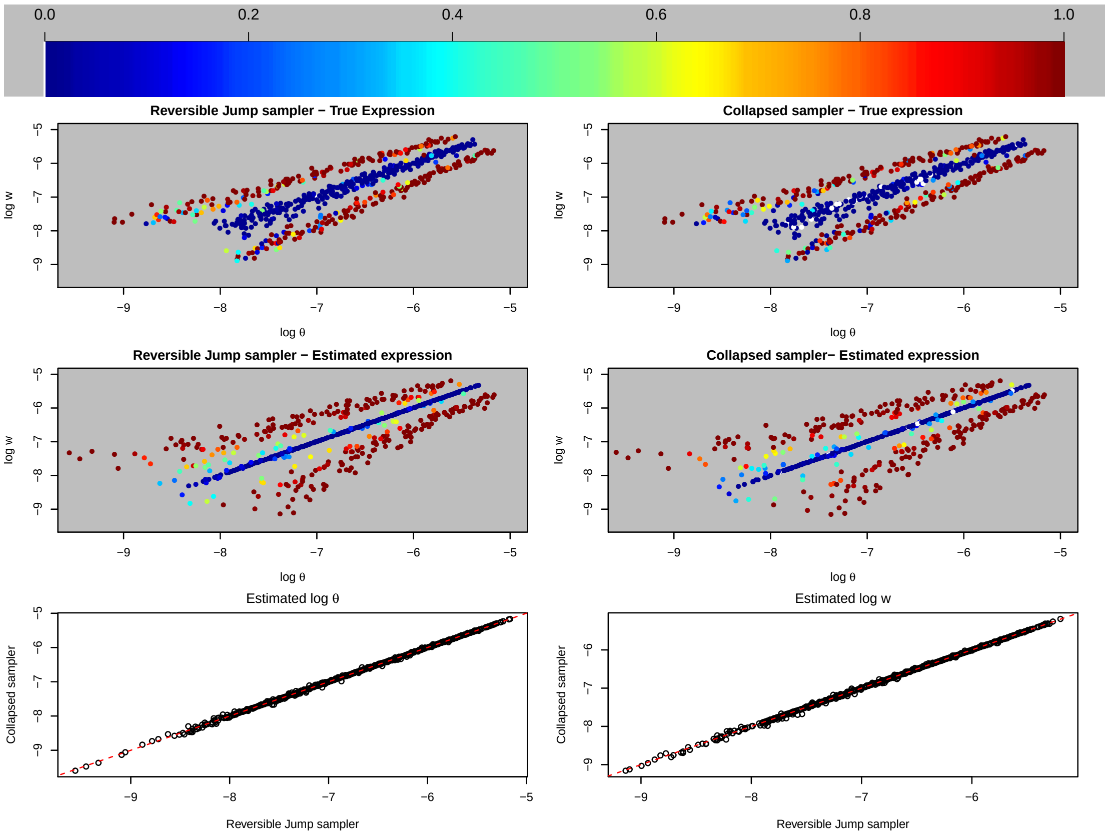

Figure 4: True log-relative expression values for the toy example. The colour corresponds to the posterior probability of differential expression according to each sampler under the Jeffreys' prior (blue, green and red colors denote values close to 0, 0.5 and 1 respectively).

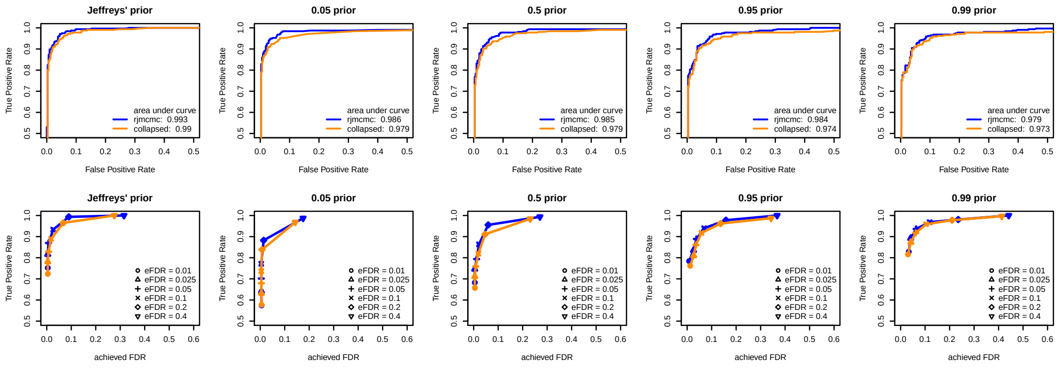

Figure 5: ROC curves (up) and power-to-achieved FDR (down) for the toy example using different prior distribution on the probability of differential expression.

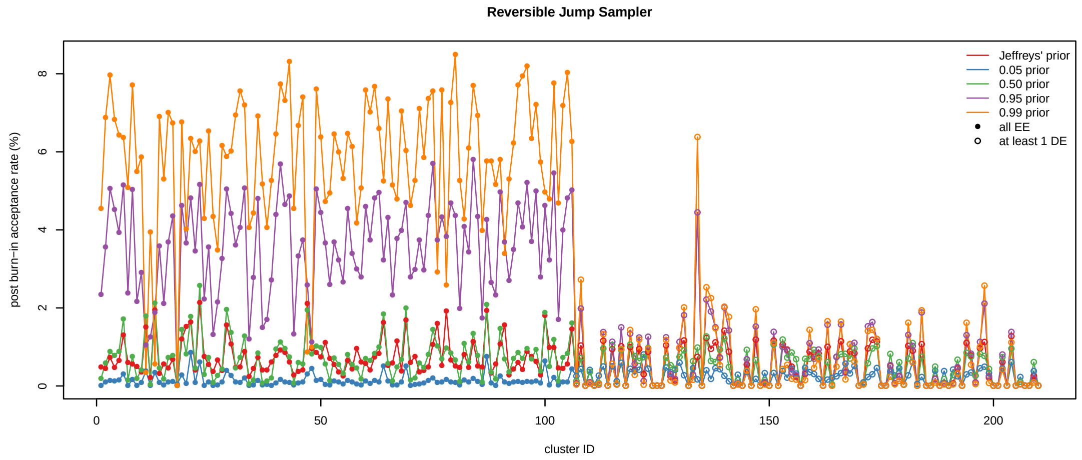

Figure 6: Reversible Jump proposal acceptance rates per cluster (after discarding the MCMC draws which correspond to the burn-in period) for the update of $c, \boldsymbol{v}$ using different prior distributions. Note that the points are reordered so that clusters exclusively consisting of (truely) EE transcripts are shown first (solid points) followed by the clusters which contain at least one (truely) DE transcript (circles).

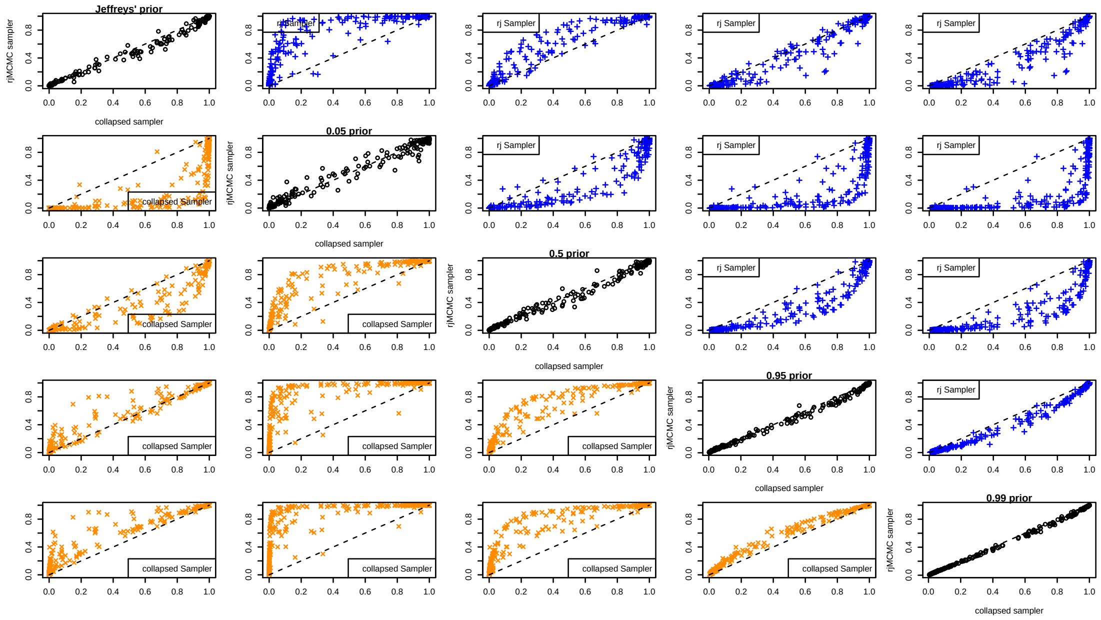

Figure 7: Prior sensitivity of the rjMCMC (blue) and collapsed (orange) sampler. The main diagonal contains scatterplots of the posterior probability of DE between the rjMCMC and collapsed samplers for each prior distribution. The scatterplots of the same posterior probabilities for all possible prior combinations per sampler is shown at the upper (rjMCMC) and lower (collapsed) diagonal.

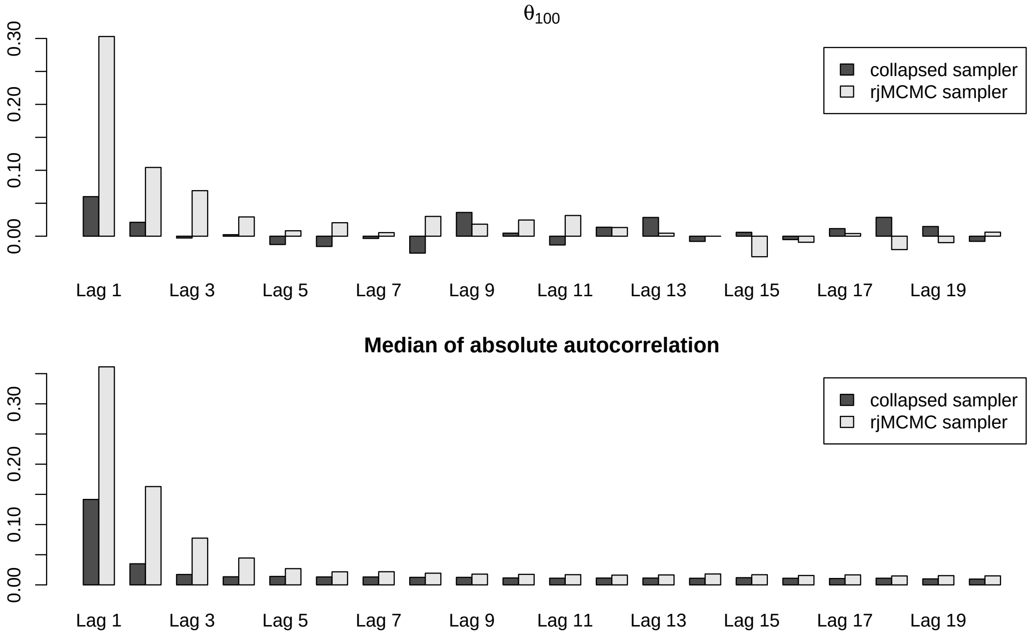

Figure 8: Top: estimated autocorrelation function of the collapsed and rjMCMC algorithm for the sampled values of $\log \theta_{100}$. Bottom: Median of absolute autocorrelations for $\log \theta_k$; $k = 1, \ldots, 630$.

We simulated approximately 300000 reads per sample, which arise from a set of $K = 630$ transcripts. The true values of the mixture weights used for the simulation are shown in Figure 4. Almost half of transcripts are Differentially Expressed and they correspond to the points that diverge from the identity line. The colour of each point corresponds to the posterior probability of differential expression for each sampler using the Jeffreys' prior distribution. The corresponding ROC curves for each sampler are shown in Figure 5, using also different prior distributions on the probability of differential expression. We conclude that the rjMCMC sampler tends to achieve higher true positive rate and a larger area under the curve. The achieved false discovery rates are shown at the second of 4. Compared to their expected values (eFDR) we conclude that both samplers achieve to control the False Discovery Rate at the desired levels, even when the prior favours DE transcripts (0.95 prior).

We also examine the acceptance rate of the reversible jump proposal, shown in Figure 6. Overall, there is a small acceptance rate of proposed moves and there is a notable increase when the prior favours DE transcripts (0.95 or 0.99 prior). This mainly affects clusters consisting exclusively of EE transcripts. This conservative behaviour of rjMCMC sampler may indicates that the mixing of the algorithm is poor for the case of EE transcripts.

Next, we compare the autocorrelation function between the two samplers when using the Jeffrey's prior distribution for the probability of DE. A typical behaviour is shown in Figure 8 (top), displaying the autocorrelation function of $\log \theta_k$ for a single transcript ($k = 100$). In order to summarize the behaviour of autocorrelations across all $K = 630$ transcripts we have computed the median of absolute autocorrelations for all θ_k; $k = 1, \ldots, K$, as shown at the bottom of Figure 8. We conclude that the mixing of the collapsed sampler is notably better.

Finally we perform a comparison between the raw sampler (that is, taking into account the

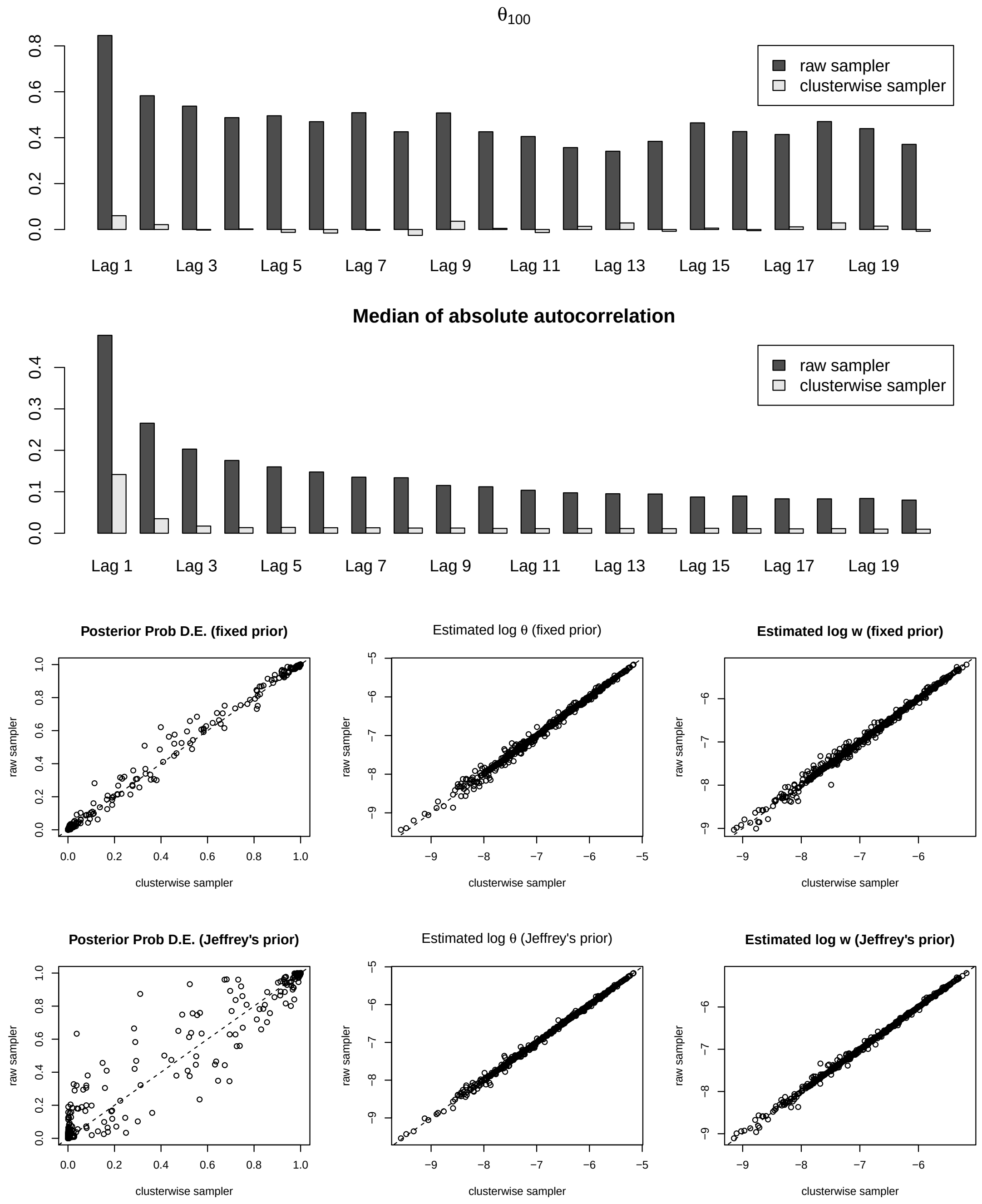

Figure 9: Comparison of the clusterwise and raw MCMC algorithm. First row: estimated autocorrelation function for the sampled values of $\log \theta_{100}$. Second row: Median of absolute autocorrelations for $\log \theta_k$; $k = 1, \ldots, 630$. Third row: Comparison of estimated posterior means.

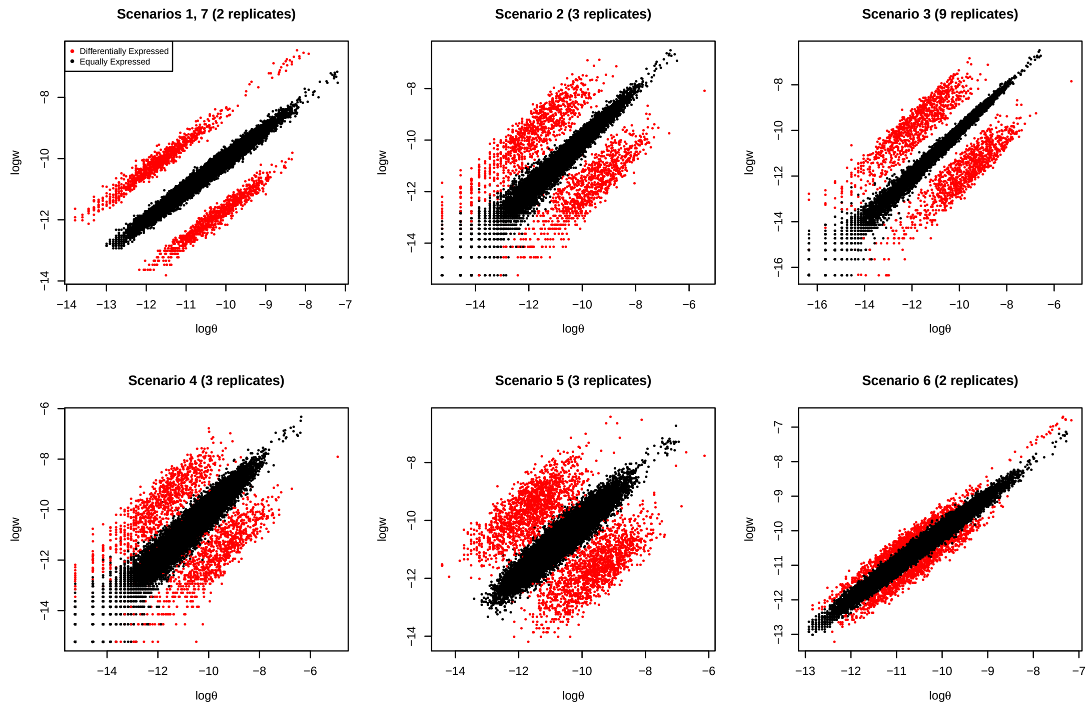

Figure 10: Logarithm of true relative expression levels for seven simulation scenarios, averaged across the corresponding number of replicates.

whole set of reads and transcripts) and the clusterwise one. For this reason we have run the raw collapsed MCMC sampler with a fixed prior of DE (equal to 0.5) as well as the Jeffrey's prior. As shown in Figure 9 (first two rows), the raw MCMC sampler exhibits very large autocorrelations compared to the clusterwise sampler (the autocorrelation function is nearly identical for both prior choices). The resulting estimates of DE and posterior means of transcript expression are shown in the third and fourth row of 9. Note that the estimates of posterior probability of DE exhibit larger variability under the Jeffrey's prior. In both cases, the transcript expression estimates exhibit strong agreement. The number of iterations of the raw sampler was set to 2000000, following a burn-in period of 200000 iterations. Such a large number of iterations in general will not be sufficient in cases that the number of transcripts grows to typical values of RNA-seq datasets, hence running the raw MCMC sampler becomes prohibitive in general cases.

K Simulation study details

In the sequel, $\mathcal{P}$ and $\mathcal{NB}(\mu, \phi)$ denote the Poisson and Negative Binomial distributions respectively, where for the latter the parameterization with mean equal to μ and variance equal to $\mu + \mu^2/\phi$ is used, $\mu \geqslant 0$, $\phi > 0$. Finally, let $\mathrm{RPK}_{jk}^{(A)}$ and $\mathrm{RPK}_{jk}^{(B)}$ denote the rpk values for transcript k at replicate j of condition A and B, respectively.

Scenario 1 (2 Poisson replicates per condition) Reads are simulated according to the following generative process.

$$
\begin{aligned}
\mu_k &= 65, \quad k = 1, \ldots, K, \quad n_d = 2g, \quad g = 1000 \\
\{k_1, \ldots, k_{2g}\} &: \text{random sample of indices (without replacement)} \subseteq \{1, \ldots, K\} \\
\delta_k^{(1)} &= 0.65, \quad k = k_1, \ldots, k_g \\
\delta_k^{(2)} &= 3.25, \quad k = k_{g+1}, \ldots, k_{2g} \\
(\mu_k^{(A)}, \mu_k^{(B)}) &= \begin{cases} (1,1)\mu_k, & k \neq k_1, \ldots, k_{n_d} \\ \left(\frac{1}{\delta_k^{(1)}}, \frac{1}{\delta_k^{(2)}}\right)\mu_k & k = k_1, \ldots, k_g \\ \left(\frac{1}{\delta_k^{(2)}}, \frac{1}{\delta_k^{(1)}}\right)\mu_k, & k = k_{g+1}, \ldots, k_{2g} \end{cases} \\
\mathrm{RPK}_{jk}^{(A)} &\sim \mathcal{P}(\mu_k^{(A)}), \quad \mathrm{RPK}_{jk}^{(B)} \sim \mathcal{P}(\mu_k^{(B)}), \quad k = 1, \ldots, K, j = 1, 2.
\end{aligned}
$$

The rpk values determined by this scenario used as input in Spanki and ≈ 2400000 reads per replicate are simulated (≈ 9600000 reads in total). For non-differentially expressed transcripts, rpk values are simulated from a Poisson distribution with mean equal to 65 for both replicates of each condition. Next, $n_d = 2000$ differentially expressed transcripts simulated with mean fold changes equal to $\mu_k^{(A)}/\mu_k^{(B)} = 1/5$, $k = 1, \ldots, g$ and $\mu_k^{(A)}/\mu_k^{(B)} = 5$, $k = g+1, \ldots, 2g$. More specifically, rpk values generated either from the $\mathcal{P}(20)$ or $\mathcal{P}(100)$ distribution. The averaged relative log-expression based on the true values are shown in Figure 10 and the points close to the identity line correspond to 26763 no-DE transcripts. The rest 2000 points that are far away from the identity line correspond to the DE transcripts. Apparently, this scenario corresponds to a clear cut case of separation between DE and non-DE transcripts at the two conditions.

Scenario 2 (3 Negative Binomial replicates per condition) Reads are simulated according to the following generative process.

$$
\begin{aligned}
\mu_k &\sim \mathcal{U}(0, 70), \quad k = 1, \ldots, K, \quad n_d = 2g, \quad g = 1000 \\
\{k_1, \ldots, k_{2g}\} &: \text{random sample of indices (without replacement)} \subseteq \{1, \ldots, K\} \\
\delta_k &\sim \mathcal{U}(\sqrt{3}, \sqrt{5}), \quad k = k_1, \ldots, k_g \\
(\mu_k^{(A)}, \mu_k^{(B)}) &= \begin{cases} (1,1)\mu_k, & k \neq k_1, \ldots, k_{n_d} \\ (\delta_k, 1/\delta_k)\mu_k, & k = k_1, \ldots, k_g \\ (1/\delta_k, \delta_k)\mu_k, & k = k_{g+1}, \ldots, k_{2g} \end{cases} \\
\mathrm{RPK}_{jk}^{(A)} &\sim \mathcal{NB}(\mu_k^{(A)}, 50), \quad \mathrm{RPK}_{jk}^{(B)} \sim \mathcal{NB}(\mu_k^{(B)}, 50), \quad k = 1, \ldots, K, j = 1, 2, 3.
\end{aligned}
$$

The rpk values determined by this scenario used as input in Spanki and ≈ 1335000 reads per replicate are simulated (≈ 8010000 reads in total). For non-differentially expressed transcripts, rpk values are simulated from the $\mathcal{NB}(65, 50)$ for all three replicates of each condition. Next, $n_d = 2000$ differentially expressed transcripts simulated with mean fold changes varying in the $\mu_k^{(A)}/\mu_k^{(B)} \in (3, 5)$, $k = 1, \ldots, g$ and $\mu_k^{(A)}/\mu_k^{(B)} \in (1/5, 1/3)$, $k = g+1, \ldots, 2g$. The averaged relative log-expression based on the true values are shown in Figure 10 and the points close to the identity line correspond to 26763 no-DE transcripts. The rest 2000 points correspond to the DE transcripts. Compared to Scenario 1, this case exhibits less separation between DE and non-DE transcripts at the two conditions due to (a) smaller fold changes, (b) increased replicate variability due to the Negative Binomial distribution and (c) larger range of transcript expression values.

Scenario 3 (9 Negative Binomial replicates per condition) The generative process is the same as Scenario 2 but with three times larger number of replicates per condition. In total ≈ 24030000 reads simulated. The averaged relative log-expression based on the true values are shown in Figure 10. Compared to Scenario 2, there should be more signal in the data in order to detect changes in expression due to the increased number of replicates.

Scenario 4 (3 Negative Binomial replicates per condition, enhanced inter-replicate variance) The generative process and the number of simulated reads is the same as Scenario 2 but with larger levels of variability among replicates. In particular we set $\phi = 10$, corresponding to five times larger variability compared to Scenario 2. The averaged relative log-expression based on the true values are shown in Figure 10. Compared to Scenario 2, there should be more uncertainty in the data in order to detect changes in expression due to the increased number of replicates.

Scenario 5 (3 Negative Binomial replicates per condition, enhanced inter-replicate variance, smaller range for the mean) The generative process and the number of simulated reads is the same as Scenario 4 but with more concentrated levels for the mean of true rpk values among replicates. In particular, we set

$$\begin{aligned}
\mu_k &= 60, \quad k = 1, \ldots, K, \quad n_d = 2g, \quad g = 1000 \\
\{k_1, \ldots, k_{2g}\} &: \text{random sample of indices (without replacement)} \subseteq \{1, \ldots, K\} \\
\delta_k &\sim \mathcal{U}(\sqrt{3}, \sqrt{5}), \quad k = k_1, \ldots, k_g \\
(\mu_k^{(A)}, \mu_k^{(B)}) &= \begin{cases} (1,1)\mu_k, & k \neq k_1, \ldots, k_{n_d} \\ (\delta_k, 1/\delta_k)\mu_k, & k = k_1, \ldots, k_g \\ (1/\delta_k, \delta_k)\mu_k, & k = k_{g+1}, \ldots, k_{2g} \end{cases} \\
\mathrm{RPK}_{jk}^{(A)} &\sim \mathcal{NB}(\mu_k^{(A)}, 10), \quad \mathrm{RPK}_{jk}^{(B)} \sim \mathcal{NB}(\mu_k^{(B)}, 10), \quad k = 1, \ldots, K, j = 1, 2, 3.
\end{aligned}$$

Note that the difference with Scenario 2 is that now μ_k has a constant value for all $k = 1, \ldots, K$ and that the selected value of ϕ results to five times higher dispersion. The averaged relative log-expression based on the true values are shown in Figure 10. It is obvious that now the range of relative expression values is smaller compared to Scenarios 2,3 and 4.

Scenario 6 (2 Poisson replicates per condition, small fold change) This is a revision of Scenario 1 under a smaller fold change between DE and EE transcripts. In this case we set $\delta_k^(1) = 65/80$ and $\delta_k^(2) = 65/50$, resulting to a fold change of 1.6 for DE transcripts (instead of 5 as used at Scenario 1). As shown in Figure 10, the classification of DE and EE transcripts is not obvious.

Scenario 7 (2 Poisson replicates per condition, unequal total number of reads) This is a revised version of Scenario 1 under different sample sizes between the two conditions. Now the first condition contains approximately 46% larger amount of data than the second one. In particular, we simulated 2.81 and 1.93 million reads per replicate of the first and second condition, respectively. However, the relative expression levels are the same as in Scenario 1, as shown at the first plot of Figure 10.

Figure 11 displays the correlation between the true configuration of DE and EE transcripts and the estimated classification per method at the 0.05 level. Note that our collapsed sampler is ranked as the best method on every scenario. Moreover, our rjMCMC sampler is marginally

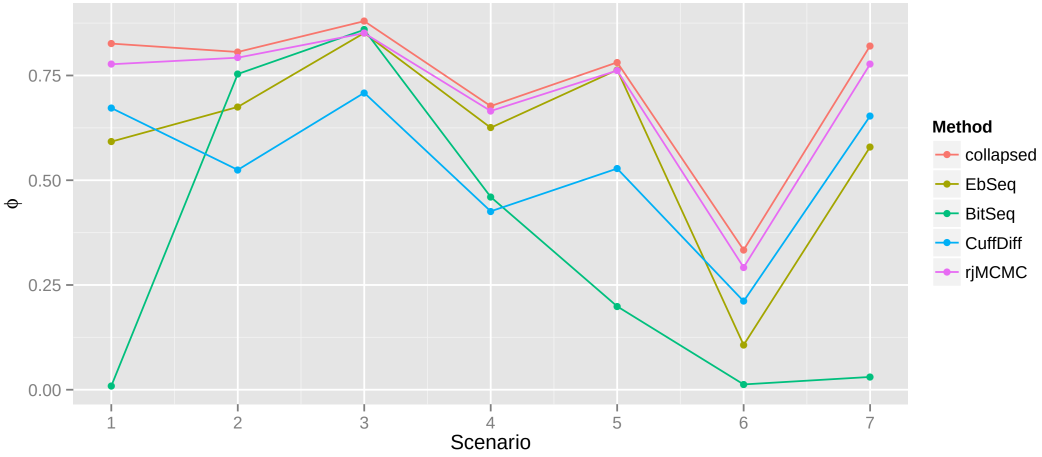

Figure 11: ϕ-coefficient between ground truth of DE and EE transcripts and the inferred classifications per method at the 0.05 level, for each simulation scenario.

the second best method. An interesting remark is that methods that control the false discovery rate exhibit a similar pattern across different scenarios, something that it is not the case for the standard BitSeq implementation. However note the improvement of standard BitSeq performance when the number of replicates is larger than two.

Figure 12 displays the ROC curves (left) and the true positive rate versus the achieved false discovery rate for the rjMCMC and collapsed samplers. The continuous lines correspond to the Jeffrey's prior while the dashed lines correspond to a fixed probability of DE (equal to 0.5). The results are essentially the same for most scenarios. A notable difference is observed at Scenario 6 where we conclude the superior performance of our method under the Jeffrey's prior.

L Implementation of the algorithm

At first, the short reads (.fastq files) for each condition (A and B) are mapped to the reference transcriptome using Bowtie. The alignments (.sam files) are pre-processed using the `parseAlignment` command of BitSeq in order to compute the alignment probabilities for each read (.prob files). These files are used as the input of the proposed algorithm in order to (a) compute the clusters of reads and transcripts and (b) run the MCMC algorithm for each cluster. The output is a file containing the estimates of relative transcript expression for each condition and the posterior probability of differential expression.

Assume that there are two replicates per sample consisting of paired-end reads: `A1_1.fastq`, `A1_2.fastq`, `A2_1.fastq` and `A2_2.fastq` for sample A and `B1_1.fastq`, `B1_2.fastq`, `B2_1.fastq` and `B2_2.fastq` for sample B. Denote by `reference.fa` the fasta file with the transcriptome annotation. Let `outputRJ` and `outputCollapsed` denote the output directory of the rjMCMC and collapsed samplers, respectively. The following code describes a typical implementation of the whole pipeline, assuming that all input files are in the working directory (replace by the full paths otherwise).

```
# build bowtie2 indices and align reads
bowtie2-build -f reference.fa reference
```

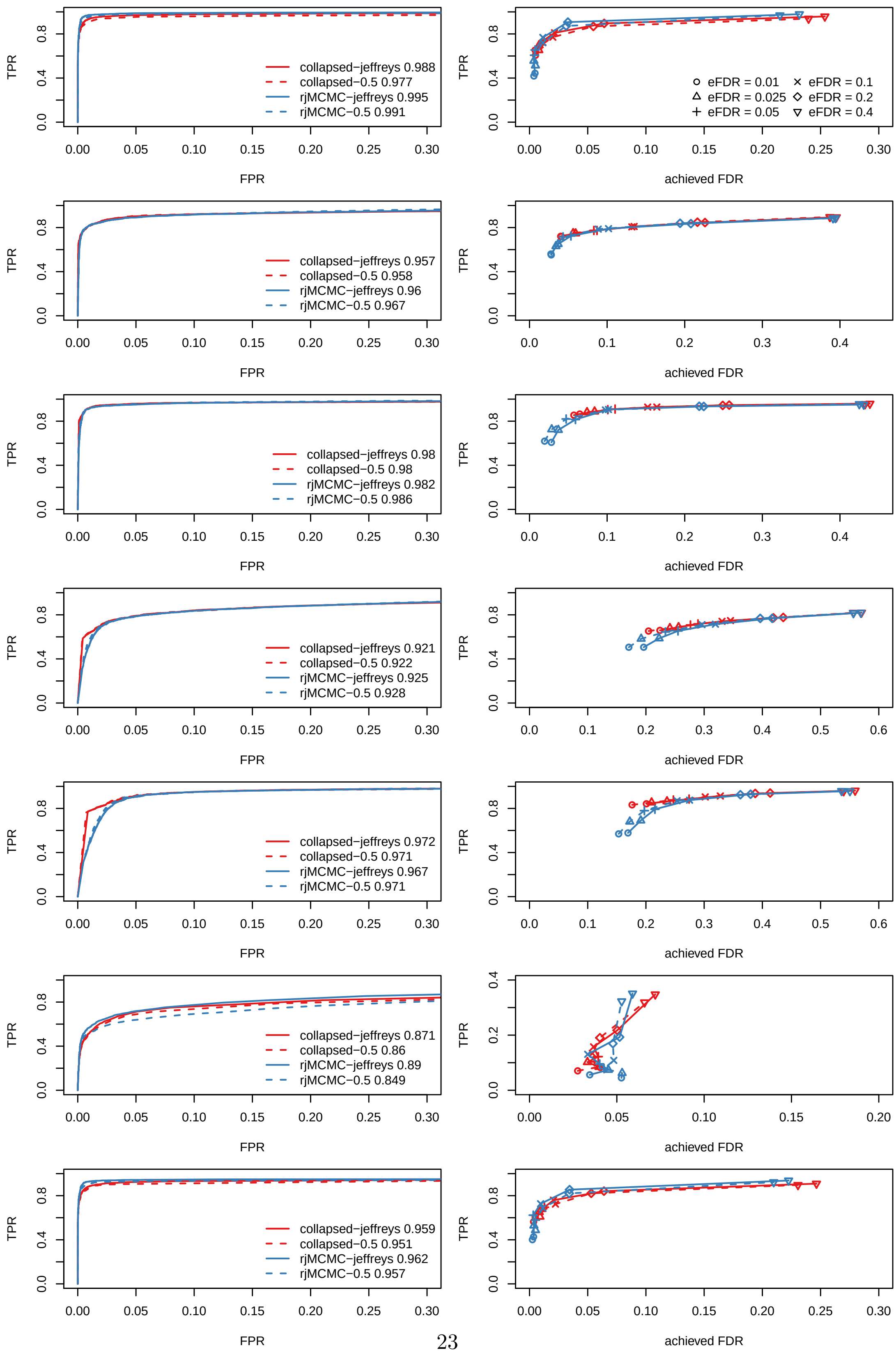

Figure 12: ROC curves (left) and power - to achieved plots (right) per simulation scenario, using different prior distribution on the probability of differential expression.

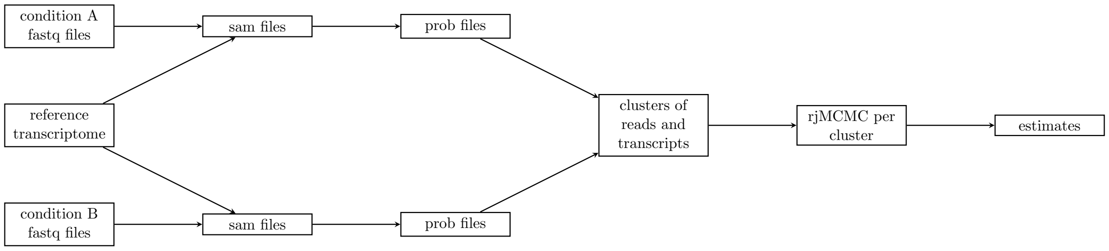

Figure 13: General work-flow of the algorithm.

```
bowtie2 -q -k 100 --no-mixed --no-discordant -x reference
                        -1 A1_1.fastq -2 A1_2.fastq -S A1.sam
bowtie2 -q -k 100 --no-mixed --no-discordant -x reference
                        -1 A2_1.fastq -2 A2_2.fastq -S A2.sam
bowtie2 -q -k 100 --no-mixed --no-discordant -x reference
                        -1 B1_1.fastq -2 B1_2.fastq -S B1.sam
bowtie2 -q -k 100 --no-mixed --no-discordant -x reference
                        -1 B2_1.fastq -2 B2_2.fastq -S B2.sam

# compute alignment probabilities with BitSeq
parseAlignment A1.sam -o A1.prob --trSeqFile reference.fa
                                                      --uniform
parseAlignment A2.sam -o A2.prob --trSeqFile reference.fa
                                                      --uniform
parseAlignment B1.sam -o B1.prob --trSeqFile reference.fa
                                                      --uniform
parseAlignment B2.sam -o B2.prob --trSeqFile reference.fa
                                                      --uniform
# compute clusters and apply the rjMCMC sampler
rjBitSeq outputRJ A1.prob A2.prob C B1.prob B2.prob
# compute clusters and apply the collapsed sampler
cjBitSeq outputCollapsed A1.prob A2.prob C B1.prob B2.prob
```

The output of the rjMCMC and collapsed samplers is written to `outputRJ/estimates.txt` and `outputCollapsed/estimates.txt`, respectively. The overall work-flow is summarized in Figure 13.

M Additional tables and figures

Table 1 illustrates the correlation between the resulting classifications for the two real datasets in Section 3.3. Table 2 reports the running time needed for our experiments using 8 threads. The run-times reported for our method contains both cluster discovery and MCMC sampling. It should be mentioned that a significant portion of the reported run-times is allocated to the clustering part which is not optimized for speed ($20\% - 35\%$ and $40\% - 45\%$ for the rjMCMC and collapsed samplers, respectively). More details regarding the computing time and memory usage demanded by our method are shown in Figure 14.

Table 1: ϕ-coefficient between the resulting classifications at the 0.05 level for HiSeq (lower diagonal) and MiSeq (upper) data.

Method	*cuffdiff*	*BitSeq*	*EBSeq*	*cjBitSeq*
cuffdiff	1	0.43	0.32	0.32
BitSeq	0.64	1	0.58	0.59
EBSeq	0.52	0.61	1	0.70
cjBitSeq	0.56	0.63	0.75	1

Table 2: Approximate total number of reads (in millions) and run-time in hours for each example.

dataset	*reads*	*cufflinks*	*BitSeq*	*rsem/EBSeq*	*rjMCMC*	*collapsed*
scenario 1	9.4	0.9	4.4	2.2	4.8	2.7
scenario 2	8.0	0.8	3.3	1.8	4.5	3.4
scenario 3	24.0	2.1	9.1	6.2	9.8	8.4
scenario 4	8.0	0.9	3.4	2.5	4.4	3.2
scenario 5	14.0	1.1	9.7	3.7	6.6	5.1
scenario 6	9.4	0.8	4.5	1.9	4.3	2.6
scenario 7	9.5	0.7	5.8	1.7	4.1	2.5
MiSeq	21.3	1.0	4.8	2.4	6.8	3.9
HiSeq	97.0	2.4	22.3	11.1	26.1	19.8

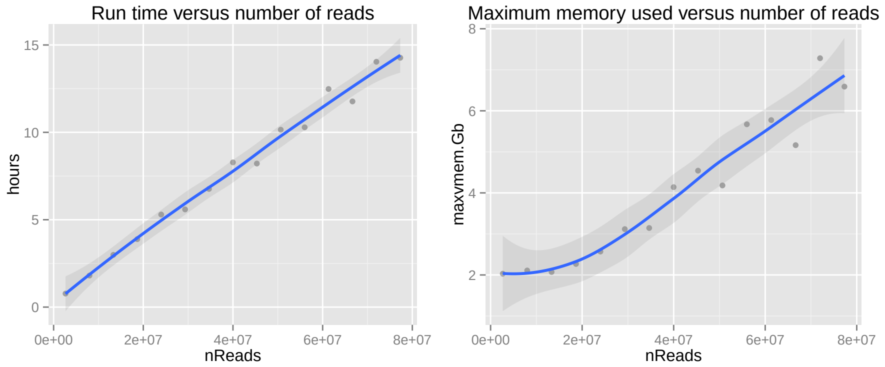

Figure 14: Run time of the algorithm (left) and maximum virtual memory used (right) versus total number of (mapped) reads corresponding to the collapsed algorithm using 12 cores.
